# Molecular Mechanisms Generating and Stabilizing Terminal 22q13 Deletions in 44 Subjects with Phelan/McDermid Syndrome

**DOI:** 10.1371/journal.pgen.1002173

**Published:** 2011-07-14

**Authors:** Maria Clara Bonaglia, Roberto Giorda, Silvana Beri, Cristina De Agostini, Francesca Novara, Marco Fichera, Lucia Grillo, Ornella Galesi, Annalisa Vetro, Roberto Ciccone, Maria Teresa Bonati, Sabrina Giglio, Renzo Guerrini, Sara Osimani, Susan Marelli, Claudio Zucca, Rita Grasso, Renato Borgatti, Elisa Mani, Cristina Motta, Massimo Molteni, Corrado Romano, Donatella Greco, Santina Reitano, Anna Baroncini, Elisabetta Lapi, Antonella Cecconi, Giulia Arrigo, Maria Grazia Patricelli, Chiara Pantaleoni, Stefano D'Arrigo, Daria Riva, Francesca Sciacca, Bernardo Dalla Bernardina, Leonardo Zoccante, Francesca Darra, Cristiano Termine, Emanuela Maserati, Stefania Bigoni, Emanuela Priolo, Armand Bottani, Stefania Gimelli, Frederique Bena, Alfredo Brusco, Eleonora di Gregorio, Irene Bagnasco, Ursula Giussani, Lucio Nitsch, Pierluigi Politi, Maria Luisa Martinez-Frias, Maria Luisa Martínez-Fernández, Nieves Martínez Guardia, Anna Bremer, Britt-Marie Anderlid, Orsetta Zuffardi

**Affiliations:** 1 Cytogenetics Laboratory, Scientific Institute E. Medea, Bosisio Parini, Italy; 2 Molecular Biology Laboratory, Scientific Institute E. Medea, Bosisio Parini, Italy; 3 Biologia Generale e Genetica Medica, Università di Pavia, Pavia, Italy; 4 Laboratorio di Diagnosi Genetica, Istituto Scientifico Oasi Maria Santissima, Troina, Italy; 5 Clinic of Medical genetics, IRCCS Auxologico Italiano, Milano, Italy; 6 Medical Genetics Section, Department of Clinical Pathophysiology, University of Florence, Firenze, Italy; 7 Medical Genetics Unit, Meyer Children's University Hospital, Firenze, Italy; 8 Pediatric Neurology Unit, Children's Hospital A. Meyer, University of Florence, Firenze, Italy; 9 Biologia Molecolare e Citogenetica, Diagnostica e Ricerca, Ospedale San Raffaele, Milano, Italy; 10 Department of Child Neuropsychiatry and Neurorehabilitation 1, Scientific Institute E. Medea, Bosisio Parini, Italy; 11 Neurofisiologia Clinica, Scientific Institute E. Medea, Bosisio Parini, Italy; 12 Department of Child Psychopathology, Scientific Institute E. Medea, Bosisio Parini, Italy; 13 Unità Operativa di Pediatria e Genetica Medica, IRCCS Oasi Maria Santissima Troina, Italy; 14 Unità Operativa di Genetica Medica, Dipartimento Materno-Infantile AUSL Imola, Imola, Italy; 15 Genetics and Molecular Medicine Unit, University of Florence, Firenze, Italy; 16 Unità Operativa di Pediatria e Neonatologia, Ambulatorio di Genetica Clinica, Ospedale S. Maria Annunziata, Firenze, Italy; 17 Developmental Neurology Unit, Carlo Besta Foundation Neurological Institute, Milano, Italy; 18 Laboratorio di Patologia Clinica e Genetica Medica, Servizio di Citogenetica, Carlo Besta Foundation Neurological Institute, Milano, Italy; 19 Unità Operativa Complessa Neuropsichiatria Infantile, Azienda Ospedaliera Universitaria Integrata Policlinico G.B. Rossi, Verona, Italy; 20 Child Neuropsychiatry Unit, Department of Experimental Medicine, University of Insubria, Varese, Italy; 21 Biologia e Genetica, Dipartimento di Scienze Biomediche Sperimentali e Cliniche, Università dell'lnsubria, Varese, Italy; 22 Medical Genetic Section, Department of Experimental and Diagnostic Medicine, University of Ferrara, Ferrara, Italy; 23 Unità Operativa Complessa Genetica Medica, Azienda Ospedaliera BBM, Reggio Calabria, Italy; 24 Service de Médecine Génétique, Département de Médecine Génétique et de Laboratoire, Höpital Cantonal de Genève, Geneva, Switzerland; 25 Department of Genetics, Biology and Biochemistry, University of Torino, Torino, Italy; 26 Struttura Complessa NPI Ospedale Martini, Torino, Italy; 27 Laboratorio di Genetica Medica, Ospedali Riuniti di Bergamo, Bergamo, Italy; 28 Department of Cellular and Molecular Biology and Pathology, “Federico II” University, Napoli, Italy; 29 Department of Health Sciences, University of Pavia, Pavia, Italy; 30 Facultad de Medicina, Universidad Complutense de Madrid, Madrid, Spain; 31 Centro de Investigación sobre Anomalías Congénitas (CIAC), Instituto de Salud Carlos III, Madrid, Spain; 32 Centro de Investigación Biomédica en Red de Enfermedades Raras (CIBERER), Madrid, Spain; 33 Servicio de Neonatología, Hospital Severo Ochoa Leganes, Madrid, Spain; 34 Department of Molecular Medicine and Surgery, Karolinska Institute, Stockholm, Sweden; 35 Fondazione IRCCS C. Mondino, Pavia, Italy; University of Pennsylvania, United States of America

## Abstract

In this study, we used deletions at 22q13, which represent a substantial source of human pathology (Phelan/McDermid syndrome), as a model for investigating the molecular mechanisms of terminal deletions that are currently poorly understood. We characterized at the molecular level the genomic rearrangement in 44 unrelated patients with 22q13 monosomy resulting from simple terminal deletions (72%), ring chromosomes (14%), and unbalanced translocations (7%). We also discovered interstitial deletions between 17–74 kb in 9% of the patients. Haploinsufficiency of the *SHANK3* gene, confirmed in all rearrangements, is very likely the cause of the major neurological features associated with PMS. *SHANK3* mutations can also result in language and/or social interaction disabilities. We determined the breakpoint junctions in 29 cases, providing a realistic snapshot of the variety of mechanisms driving non-recurrent deletion and repair at chromosome ends. *De novo* telomere synthesis and telomere capture are used to repair terminal deletions; non-homologous end-joining or microhomology-mediated break-induced replication is probably involved in ring 22 formation and translocations; non-homologous end-joining and fork stalling and template switching prevail in cases with interstitial 22q13.3. For the first time, we also demonstrated that distinct stabilizing events of the same terminal deletion can occur in different early embryonic cells, proving that terminal deletions can be repaired by multistep healing events and supporting the recent hypothesis that rare pathogenic germline rearrangements may have mitotic origin. Finally, the progressive clinical deterioration observed throughout the longitudinal medical history of three subjects over forty years supports the hypothesis of a role for *SHANK3* haploinsufficiency in neurological deterioration, in addition to its involvement in the neurobehavioral phenotype of PMS.

## Author Summary

Terminal chromosome deletions are among the most commonly observed rearrangements detected by cytogenetics and may result in several well-known genetic syndromes. We used 22q13 deletions to study how these types of chromosome abnormalities arise. Children with Phelan/McDermid syndrome, caused by deletion of the terminal portion of chromosome 22, experience developmental delay, absent or severely delayed speech, and frequent behavioral problems. Lack of one copy of *SHANK3*, a key gene for the correct development and organization of brain synapses, is very likely the basis of the syndrome's major neurological features. Deletion of additional genes probably causes more complex phenotypes in subjects with larger deletions. We also studied patients who only lack a portion of *SHANK3* and demonstrated that small, hard-to-detect deletions of this gene may cause substantial clinical problems. Until now, the 22q distal deletion had been only diagnosed in very young people. We studied a large group of patients of different ages and discovered that all adult patients face progressive cognitive decline. Our study demonstrates that deletion of the terminal portion of chromosome 22, a prototype for terminal deletions in human chromosomes, can occur in several ways. Mosaic deletions of different size can also form in early embryogenesis.

## Introduction

Deletions involving the distal portion of chromosomes are among the most commonly observed rearrangements detected by cytogenetics [1] and result in several well-known genetic syndromes such as 1p36 monosomy (MIM: 607872), Cri-du-chat (5p-, MIM: 123450), Miller-Dieker (17p-, MIM: 247200), monosomy 18q (18q-, MIM: 6011808) monosomy 9p (MIM: 158171), Wolf-Hirschhorn (4p-, MIM: #194190), 9q34.3 microdeletion (MIM: 610253), monosomy 2q37 (MIM: 600430) and Phelan-McDermid (PMS, MIM: 606232) syndromes. Over the past 15 years, technological advances in the molecular cytogenetic diagnosis of mental retardation, such as subtelomere screening and high-resolution genome analysis, have strongly enhanced the detection rate of an increasing number of chromosome rearrangements involving subtelomeric regions associated with mental retardation.

Telomere loss caused by double-strand breaks (DSBs) can generate, if not properly repaired, chromosome instability, cell senescence, and/or apoptotic cell death. Terminal deletions can be repaired and stabilized through the synthesis of a new telomere (telomere healing), demonstrated through sequence analysis of terminal deletions that showed *de novo* telomeric repeats attached to the remaining chromosomal sequences [2]–[4]; by telomerase-independent recombination-based mechanisms [5], [6]; by obtaining a telomeric sequence from another chromosome (telomere capture) resulting in derivative chromosomes [7], [8]; finally, by chromosomal circularization, leading to the formation of a ring chromosome [9], [10]. However, in spite of their relatively frequent occurrence, the molecular bases for generating and stabilizing terminal chromosome deletions in humans are still poorly understood, since the breakpoints have been analyzed at the base-pair level in only few studies [11], [12]. Questions remain about the timing of breakpoint repair, the relative importance of the above-mentioned mechanisms in terminal deletions affecting specific chromosomes, the role of repetitive elements, long terminal repeats and other DNA elements in chromosome breakage and stabilization.

In this study, we used deletions of 22q13, which represent a substantial source of human pathology [13], [14], as a model for investigating the molecular mechanisms of terminal deletions.

We characterized at the molecular level 40 new and 4 previously published subjects with 22q13 chromosome rearrangements [15], [16] aiming to identify the molecular mechanisms involved in stabilizing the deletions in patients with monosomy 22q13 and, more generally, to obtain new insight in the mechanisms underlying terminal deletions. Genotype-phenotype relationship, including the detailed clinical history of three adult patients that may help to define the lifelong outcome of PMS, is also discussed.

## Results

### Clinical profile of patients with 22q13 microdeletion syndrome

Patients included 26 females and 18 males, with ages ranging from birth to 47 years. Six patients (P25–29, P33) had a ring chromosome 22, five (P37–38, P42–44) had interstitial 22q13.3 deletions, three (P11, P15, P16) carried derivative chromosomes, while the remaining patients had terminal deletions (Table 1).

**Table 1:** Description of the rearrangements in 44 subjects with 22q13 deletions.

Patient	Gender	Ascertainment method	Karyotype	Rearrangement	Del22q13 size	Associated genome imbalance, size	Repair mechanism	Parental origin
P1	M	Tel-FISH	Del(22)(q13.3)dn	Terminal deletion	0.9 Mb		Telomere healing	Pat
P2	F	K	Del(22)(q13.31)dn	Terminal deletion	5.38 Mb		Telomere capture	Pat
P3	F	Tel-FISH	Del(22)(q13.32)dn	Terminal deletion	2.5 Mb		Telomere healing	Mat
P4	M	Tel-FISH	Del(22)(q13.32)dn	Terminal deletion	1.64 Mb		Telomere healing	U
P5	F	K	Del(22)(q13.31)dn	Terminal deletion	6.5 Mb		Telomere healing	Pat
P6	M	Tel-FISH	Del(22)(q13.32)dn	Terminal deletion	2.65 Mb		Telomere healing	Mat
P7	F	Tel-FISH	Del(22)(q13.31)dn	Terminal deletion	3.5 Mb		Telomere healing	Mat
P8	M	K	Del(22)q(13.32)dn	Terminal deletion	8.1 Mb		Telomere healing	Pat
P9	M	Tel-FISH	Del(22)(q13.31)dn	Terminal deletion	0.8 Mb			Pat
P10	F	K	Del(22)(q13.2)dn	Terminal deletion	8.1 Mb			U
P12	F	aCGH	Del(22)(q13.31)dn	Terminal deletion	4.98 Mb		Telomere healing	Pat
P13	F	aCGH	Del(22)(q13.3)dn	Terminal deletion	1.08 Mb		Telomere healing	U
P14	F	K	Del(22)q(13.31)dn	Terminal deletion	5.8 Mb		Telomere healing	U
P17	M	K	Del(22)(q13.2)dn	Terminal deletion	7.6 Mb			Pat
P18	F	Tel-FISH	Del(22)(q13.31)dn	Terminal deletion	4.7 Mb			U
P19	M	Tel-FISH	Del(22)(q13)dn	Terminal deletion	3.7 Mb			U
P20	F	K	Del(22)q(13.2q13.3)dn	Terminal deletion	7.2 Mb		Telomere healing	Pat
P21	F	Tel-FISH	Del(22)(q13.31)dn	Terminal deletion	4.7 Mb		Telomere healing	Mat
P22	F	Tel-FISH	Del(22)(q13.32)dn	Terminal deletion	1.9 Mb			Mat
P23	M	aCGH	Del(22)(q13.31)dn	Terminal deletion	3.4 Mb			Pat
P24	F	aCGH	Del(22)(q13.32)dn	Terminal deletion	1.8 Mb			Pat
P30	F	aCGH	Del(22)(q13.31)	Terminal deletion	3.4 Mb		Telomere healing	Pat
P31	M	Tel-MLPA, aCGH	Del(22)(q13.33)dn	Terminal deletion	122,392 bp		Telomere healing	Pat
P32	F	aCGH	Del(22)(q13.33)dn	Terminal deletion	122,388 bp		Telomere healing	Pat
P34	M	Tel-FISH	Del(22)(q13.31)dn	Terminal deletion	4.4 Mb		Telomere healing	Pat
P35	M	Tel-FISH	Del(22)(q13.31)dn	Terminal deletion	∼4 Mb			U
P36^b^	F	K	Del(22)(q13.2)dn mosaic 75%	Terminal deletion	9.0 Mb		Telomere healing	Pat
P39	M	aCGH	Del(22)(q13.3q13.3)	Terminal deletion	122,498 bp		Telomere capture	Pat
P40	F	K	Del(22)(q13.2q13.3)	Terminal deletion	7.4 Mb		Telomere capture	Mat
P41	F	K	Del(22)(q13.31q13.3)	Terminal deletion	5.8 Mb			Pat
P37	F	aCGH	Del(22)(q13.3q13.3)	Interstitial deletion	73,833 bp		NHEJ	Pat
P38	F	aCGH	Del(22)(q13.3q13.3)	Interstitial deletion	44,174 bp		NHEJ	Pat
P42	M	aCGH	Del(22)(q13.3q13.3)	Interstitial deletion	17,626 bp		FoSTeS	U
P43	F	aCGH	Del(22)(q13.3q13.3)	Interstitial deletion	26,914 bp		NHEJ	U
P44	M	aCGH	Del(22)(q13.3q13.3)	Interstitial deletion	38,948 bp		NHEJ	U
P25	F	K	Del(22)(q13.32)dn	Ring 22	2.16 Mb			Pat
P26	F	K	Del(22)(q13.33)dn	Ring 22	1.2 Mb		NHEJ	U
P27	M	K	Del(22)(q13.31)dn	Ring 22	5.2 Mb			Mat
P28	F	K	Del(22)q(13.33)dn	Ring 22	0.45 Mb	Dup(22)(q11–q13.23), 18 MbDup(22)(q12.3–q13.2), 4.2 Mb		Mat
P29	M	K	Del(22)(q13.31)dn mosaic 30%	Ring 22	3.2 Mb			Pat
P33	M	K	Del(22)(q13.32)dn	Ring 22	2.04 Mb			Pat
P15^a^	F	K	Del(22)(q31.31)mat	Derivative chromosome	4.3 Mb	Dup(12q24.33qter), 0.503 Mb	NHEJ	Mat
P16^a^	M	K	Del(22)(q31.31)mat	Derivative chromosome	4.3 Mb	Dup(12q24.33qter), 0.503 Mb	NHEJ	Mat
P11^a^	F	K	Del(22)(q13.31)pat	Derivative chromosome	5 Mb	Dup(12q24.32qter), 5.7 Mb	NHEJ	Pat

K: karyotype; Tel-FISH: Subtelomeric Fish analysis (Tel Vysion Vysis or Tel kit Cytocell);Tel-MLPA: MLPA analysis of the 22q subtelomeric region; aCGH: array-CGH; F: female; M: male; Mat: maternal, Pat: paternal; Mb: megabases; bp: base pairs. The total size is calculated between breakpoints or between the breakpoint and the end of chromosome 22 assembly (UCSC hg18).

a Reference [15];

b Reference [16].

We excluded from the clinical analysis patients with a derivative chromosome 22 (P11, P15, P16) and subject P28 with a complex ring 22 rearrangement, since the additional duplicated regions could complicate the assessment.

The features observed in the 40 cases in our series were compared to the characteristic features of the 22q13 deletion syndrome [17] (Table S1).

#### Clinical medical history of adult patients

Since to date old patients with 22q13.3 deletion syndrome have not been described and no longitudinal data are available to determine their life expectancy, we report the medical and clinical history of three adult patients over forty years (P10, P30 and P33) (Text S1 for additional medical details).

Subject P10:

The patient is a woman referred to a geneticist at the age of 40 years in the context of a diagnostic evaluation of people living in an institution for mentally disabled people. She presented absence of language and severe mental retardation. Facial dysmorphisms were also evident (Figure S1F–S1J). Neurological evaluation showed spastic paraparesis. At age 39, she suffered from frequent epileptic seizures, in spite of antiepileptic drugs. At age 43, she experienced very fast motor and cognitive decline; as a consequence, she was not able to stand, walk or even make eye contact anymore; her spastic tetraparesis markedly increased. Right renal agenesis was diagnosed during a control abdominal ultrasonography. She died at 47 years for renal failure while in a vegetative state.

Subject P30:

The patient is a woman referred for clinical genetics evaluation at the age of 40 years because of severe cognitive impairment and mild craniofacial dysmorphisms. She suffered from epilepsy, cortical tremor (starting at the age of 39 years) and poor speech. Minor facial dysmorphic features were observed (Figure S1A–S1E).

Subject P33:

The patient is a male first referred to a geneticist at the age of 41 years in the context of familial genetic counseling. The dysmorphological examination revealed evident aspecific dysmorphisms (Figure S1K–S1N). He presented with total absence of language, severe mental retardation, delayed motor development and microcephaly. At the age of 34 years, he developed type 2 diabetes, well compensated by oral hypoglycemic therapy, and had three spontaneous pneumothorax episodes at the upper lobe of his left lung.

#### Patients with cryptic interstitial 22q13.3 deletion disrupting the *SHANK3* gene

The clinical features of five cases with microdeletions involving only *SHANK3* (P37, P44) or *SHANK3* and *ACR* (P38, P42–43) are summarized in Table 2; their detailed medical history is described in Text S1.

**Table 2:** Clinical characteristic of PMS in subjects with interstitial 22q13 microdeletions.

Clinical characteristic	P37	P38	P42	P43	P44	Delahaye et al [41]	TOT
***Growth***							
*Normal/accelerated*	+	short stature		+	+	+	4/6
***Neurodevelopment***							
*Hypotonia*	+^a^	−	−	−	−	−	1/6
*Developmental delay*	+	+	+	+	+	+	6/6
*Delayed/absent language*	+	+	+	+	+	+	6/6
*Autism*	−	+	−	+	−	−	2/6
***Facial dysmorphisms***	+^b^	+	−	−	+^c^	+	3/6
***Extremities***							
*Large and flashy hands*	−	−	−	−	−	+	1/6
*2^nd^–3^rd^ toe syndactyly*	−	−	−	−	−	+	1/6

a abdominal hypotonia;

Facial dysmorphisms overlapping those observed in PMS:

b subject P37: wide nasal bridge, puffy cheeks, pointed chin, bulbous nose;

c subject P44: flat midface, long eyelashes, wide nasal bridge, puffy cheeks, bulbous nose, large/dysplastic ears.

### Parental origin of the deletions

The parental origin of the *de novo* 22q13 rearrangements was elucidated in 30 families (Table 3).

**Table 3:** Parental origin of the *de novo* 22q13 deletions.

Chr. 22 anomaly	Informative cases (N)	Paternal origin (%)	Maternal origin (%)
*del(22)*	25	19 (76%)	6 (24%)
*ring 22*	5	3 (60%)	2 (40%)
*Total*	30	22 (73%)	8 (27%)

The majority of terminal (17/23) and interstitial (2/2) deletions for which parental origin was available had paternal origin. Three of five ring 22 cases (60%) were also of paternal origin, while two were maternal.

### Molecular characterization of 22q13 deletions

We collected 40 new unrelated patients with 22q13 deletions and re-analyzed four previously published cases (Table 1).

Nine subjects (P2, P8, P10, P14, P17, P20, P36, P40, P41) showed a 22q13 deletion on high- resolution G- banding karyotype (550 bands); in three of them, previous low resolution banding karyotype had missed the rearrangement. Six cases showed a ring 22 at karyotype analysis. One of them (P29) was a mosaic. In one subject (P31), the presence of a terminal 22q13.3 microdeletion was first suspected in a routine subtelomere screening by multiplex ligation-dependent probe amplification (MLPA, kit P036, MRC Holland) that showed a possible deletion at the *RABL2B* locus, and subsequently diagnosed by aCGH analysis (244k, Agilent). Subtelomeric FISH screening with cosmid clones covering the distal 22q-140 kb [18] (data not shown) further confirmed the terminal 22q13.3 microdeletion with breakpoint between exons 8–9 of the *SHANK3* gene. The remaining twenty-four patients had normal karyotype results and were ascertained either through subtelomere-FISH or array-CGH analysis.

Whole-genome array-CGH using several available platforms (44k, 105k, 244k) was performed on all patients diagnosed through classical cytogenetic methods, except for subject P35, in order to determine the genomic size of the deletion and exclude any concurrent microdeletion/microduplication elsewhere in the genome.

**Figure 1:**
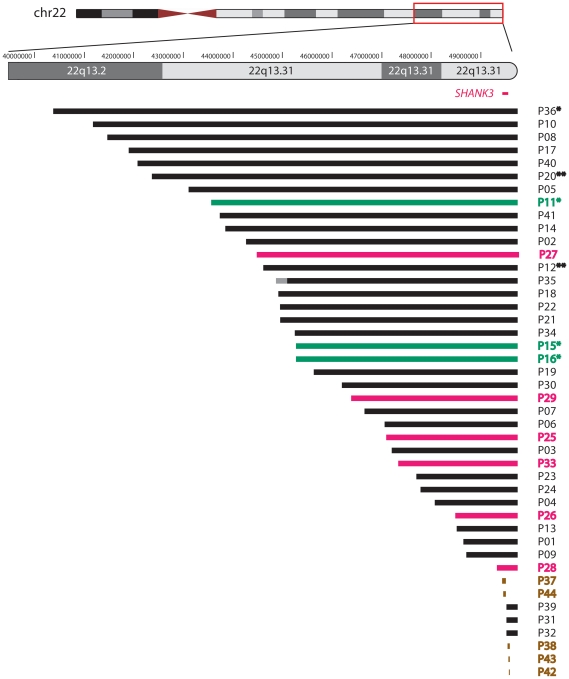
Schematic representation of the 22q13 rearrangements. An ideogram of chromosome 22 is shown at the top with genomic coordinates of the boxed terminal region of interest shown at 1 Mb intervals. The location of the *SHANK3* gene is marked in red. Each patient is represented by a horizontal line corresponding to the size of his deletion as determined by aCGH analysis. Each patient's code number is shown on the right side of the lines; asterisks (*) indicate previously published cases. Double asterisks (**) indicate mosaic deletions. The lines' colors correspond to 22q13 rearrangement categories: simple deletions are depicted in black, derivative chromosomes 22 in green, rings 22 in pink, and interstitial deletions in brown. Forty-four patients are represented; the breakpoint interval (represented in grey) in subject P35 was narrowed down to ∼400 kb by FISH analysis with BAC clones RP11-194L8 (chr22:44,951,438–45,122,714, still present) and RP11-266G21 (chr22:45,543,178–45,711,912, deleted).

This approach allowed the identification of 22q13 deletions, varying in size between 0.14 and 9.0 Mb, in 39 subjects (Figure 1). The breakpoints were scattered along the 22q13 region and no breakpoint grouping was observed. We precisely delineated the boundaries of each deletion by commercial high-resolution (244k, Agilent) or customized aCGH analysis. Further improvements in resolution, obtained with subject-specific qPCR amplification experiments, allowed the design of oligonucleotide primers to specifically amplify the junction fragments.

### Terminal deletions

We attempted to clone the deletion breakpoints of all 33 patients with apparently terminal 22q13 deletions by postulating healing of the truncated 22q sequences through the addition of a new telomere sequence at the breakpoint. Forward primers were designed proximally to each breakpoint and used for nested PCR, together with telomere-specific primers. Using this strategy, we isolated twenty-two breakpoints from 20 cases with terminal deletions (P1, P3–P8, P12–P14, P20, P21, P30–P32, P34, P36) (Figure S2). Nineteen breakpoints from 17 subjects contain 3–48 copies of the GGTTAG hexamer. Alignment of the chromosome-specific sequences flanking the telomeric repeats with the human genome reference sequence revealed the immediate proximity of the repeats to the chromosome-specific sequences in 16 breakpoints. Three breakpoints (P20 BP3, P8, P7) contain 2, 14, and 20 additional bases not present in the reference sequence, respectively. Two subjects (P31, P32) carry recurrent 22q terminal deletions [18]. The junction fragment in subject P8 contains a perfect 7-bp inverted palindrome. Thirteen of the 19 breakpoints fall inside repetitive sequences (SINE, LINE, DNA-type, simple repeats) (Figure S2). One breakpoint junction (P2) contains a (GGTGAG)_n_ repeat, fortuitously amplified because of its homology with the Tel-ACP primer, instead of the expected (GGTTAG)_n_. In a second junction (P39), the telomere sequence is preceded by (GGTCAG)_6_. A third breakpoint (P40) is joined to the terminal 450 bp of a Xp/Yp chromosome arm.

**Figure 2:**
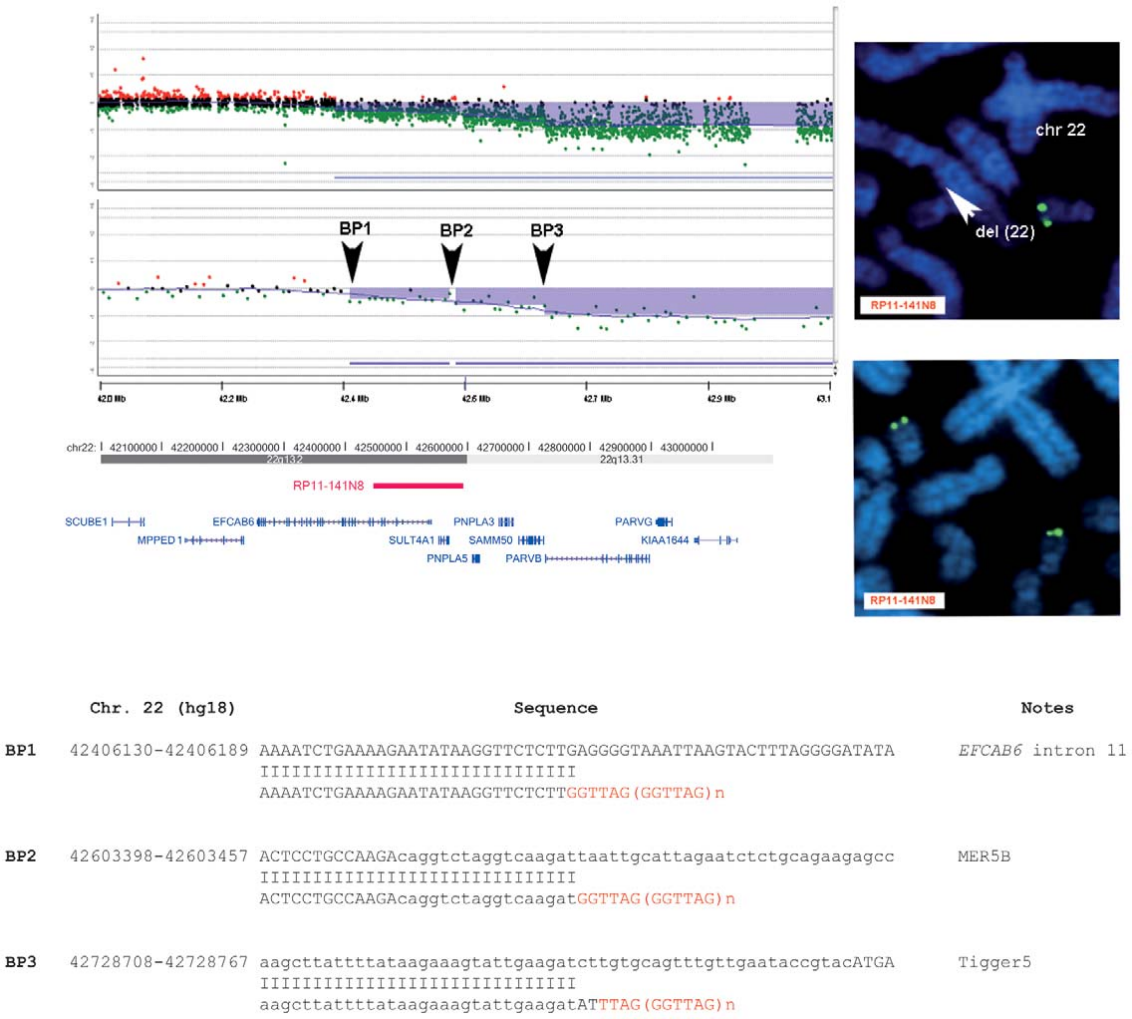
Molecular characterization of the 22q13.2 terminal deletion in subject P20. A, Magnified view of the aligned breakpoint boundaries detected by array-CGH analysis using an oligonucleotide-based custom 22q13 microarray (top) and a 180k Agilent kit (bottom); the deleted regions are shaded in blue. Arrowheads delimit two mosaic-deleted regions: the BP1–BP2 deletion region (from 42406240 to 42603381 bp) has an average log ratio of −0.3; the BP2–BP3 deletion region (from 42603381 to 42726895 bp) has an average log ratio of −0.5; the deleted region between BP3 and the telomere (from 42726895 to the end of chromosome 22) has an average log ratio of −0.8. The aligned UCSC map (hg18) is depicted at the bottom. The red bar indicates the map position of the RP11-141N8 BAC clone we used to confirm by FISH the mosaicism of the BP1–BP2 region. All genes (blue bars) mapping within the BP1–BP3 regions are shown. B, FISH analysis using the RP11-141N8 clone confirms a mosaic deletion of the BP1–BP2 region revealing: (top) the presence of hybridization signals (green signal) on only one chromosome 22 (arrowhead) in 30% of the metaphases analyzed; (bottom) the presence of hybridization signals (green signals) on both chromosome 22 homologues in the remaining 70% of the metaphases analyzed (bottom). C, Tel-ACP amplification and direct sequencing of the amplified fragments revealed the breakpoint junctions at BP1, BP2 and BP3. A telomere repeat is present at all three breakpoints.

Interestingly, high-resolution aCGH analysis allowed the identification of a patient (P20) carrying a mosaic of at least three lines with 22q13.2 terminal deletions, each with a different breakpoint (Figure 2A). All breakpoints were located in a ∼400 kb interval. FISH analysis with clone RP11-141N8 (AQ388763 at 22q13.2), positioned between BP1 and BP2 (Figure 2A, 2B) confirmed the presence of a mosaic deletion in 30% of the metaphases analyzed (Figure 2B). We cloned all three identified breakpoints: the more proximal is located in intron 11 of the *EFCAB6* gene; the intermediate falls in a MER5B repeat; the more distal in a Tigger5 repeat (Figure 2C). High-resolution aCGH profiling suggested the presence of at least two mosaic breakpoints in a second patient (P12) (Figure S3A, S3B), but we were only able to clone one of them (Figure S3C).

### Interstitial deletions

**Figure 3:**
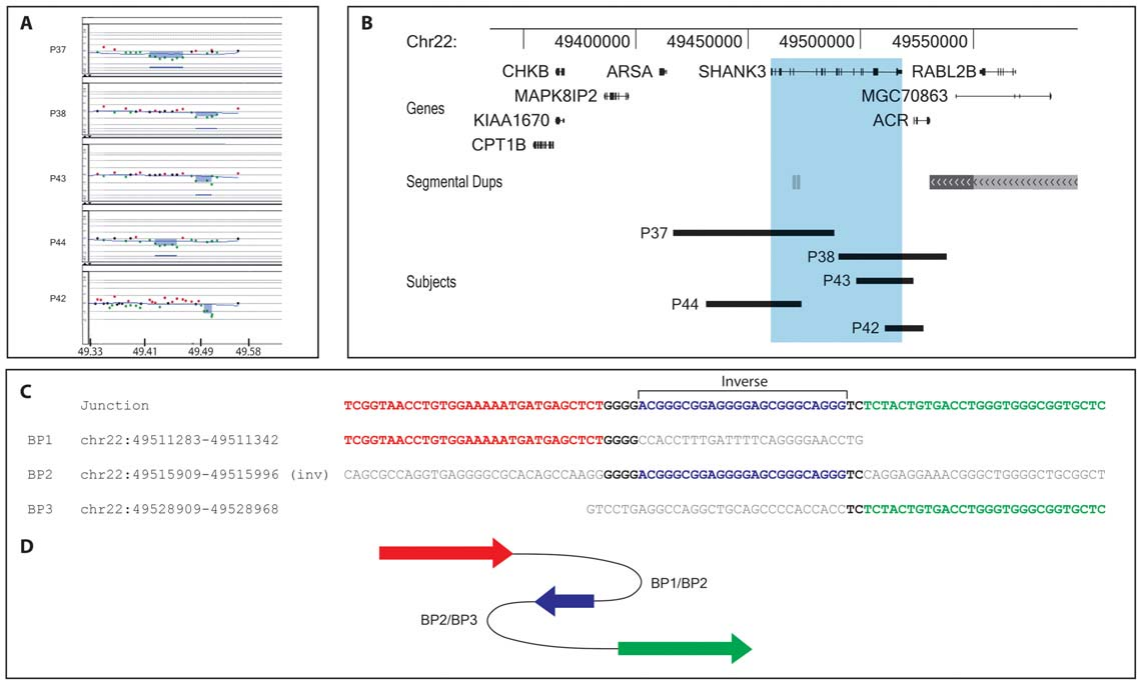
22q13.3 interstitial microdeletion detected by array-CGH analysis. A, aligned aCGH profile (P37–38, P43–44: 180k Agilent kit; P42: 244k Agilent kit) details of all interstitial deletions; the deleted regions are shaded. B, map of the distal 22q13.3 region; the deletions are represented by black bars; the region overlapping the *SHANK3* gene is shaded in light blue. All genes mapping in the region are shown. C, sequence alignment of the breakpoint junctions of subject P42 showing the homology with three genomic regions. The proximal breakpoint sequence is shown in red, the middle 24 bases in inverted orientation are blue, the distal breakpoint sequence in green; microhomologies between sequences at the breakpoints are depicted in bold. D, cartoon showing the respective position and orientation of the breakpoint sequences in P42 as arrows, colored as in C.

Our series also includes five patients with interstitial 22q13.3 deletions disrupting the *SHANK3* gene (P37, P38, P42–44) (Table 2, Figure 3A, 3B). The region distal to the deletions in P38 and P42 lies in a paralogous sequence containing the *RABL2B* gene, with almost complete identity with the chromosome 2 region containing *RABL2A*, and only one 180k/244k (Agilent) aCGH probe covers it. Subtelomere FISH screening with cosmid probes spanning the terminal 100 kb of distal 22q (data not shown) and qPCR experiments confirmed the findings. Specific amplification of the junctions by long-range PCR followed by sequencing analysis precisely defined each rearrangement's structure (Figure 3C, Figure S2).

The 74 kb interstitial deletion in P37 encompasses exons 1–17 of *SHANK3*; the 44 kb deletion in P38 covers exons 19–23 of *SHANK3* and the whole *ACR* gene; the 18 kb deletion in P42 includes exon 23 of *SHANK3* and exons 1–3 of *ACR*; the 27 kb deletion in P43 includes exons 20–23 of *SHANK3* and exons 1–3 of *ACR*; the 34 kb deletion in P44 overlaps exons 1–9 of *SHANK3* (Figure 3B). We found no homology between any proximal and distal breakpoint region. Repeated sequences (LTR and LINE) are present in three breakpoints; ten additional nucleotides were inserted at the junction of P37 (Figure S2). In P42, the breakpoint junction contains 23–29 bps identical to the reverse complement of a sequence in the middle of the deleted region; this sequence shows 4 and 2 bp microhomologies with the proximal and distal breakpoints, respectively (Figure 3C).

### Ring 22 chromosomes

Six subjects (P25–P29, P33) carry a 22q13 terminal deletion associated to ring 22 chromosome; one of them (P29) shows a mosaic deletion in 30% of the cells (not shown). We also identified a complex ring 22 rearrangement consisting of a 240 kb terminal 22q deletion, concurrent with two additional, non-contiguous, ∼18 Mb and ∼4.2 Mb 22q duplications at 22q11–q12.3 and 22q12.3–q13.2, respectively, in subject P28 (Figure S4).

The only breakpoint we were able to identify in a patient with ring 22 (P26) (Figure S5A) was cloned using inverse PCR and shows a junction between an *Alu* sequence on 22q and a repeated sequence with homology to pericentromeric and subtelomeric regions on several chromosomes (Figure S5B). There is no homology between the two breakpoints. In this case, as well as in cases P27 and P29, we verified the absence of interstitial pan-telomeric sequences with a PNA probe (Figure S5C). Unfortunately, FISH analysis could not be performed on the remaining three cases (P25, P28, P33) due to the lack of archival material.

### Unbalanced translocations

Three patients (P11 and brother/sister pair P15–16) (Cases 1, 2, and 3, respectively, in [15]) carry a derivative chromosome 22 inherited from a parent carrier of a balanced translocation.

In case P11, aCGH analysis identified the loss of a 5 Mb segment of distal 22q13.31–q13.3 and the gain of a 5.7 Mb region of chromosome 12q24.32–q24.33 (Figure S6A); the proband's father carries a balanced 12q;22q translocation. We amplified the junction between 12q24.32 and 22q13.31 by long-range PCR using a forward primer (22F) from the der(22) undeleted flanking region and a reverse primer (12R) corresponding to the 12q duplicated region. The same fragment was amplified from the carrier father but not from the mother or other control DNAs (not shown). Sequencing of the junction fragments revealed that the two breakpoints share only a 4 bp microhomology (Figure S6B). Similarly, sibs P15 and P16 both inherited the der(22) chromosome from their mother who carries a balanced 12q;22q translocation. The two sibs carry a 4.3 Mb 22q13 deletion and a 0.5 Mb 12q24.33–q24.33 duplication (Figure S6C). In these patients, the rearrangement is between an *Alu* repeat on chromosome 22q and a (TGAG)n simple repeat on chromosome 12q. The two breakpoints share only a 5-bp microhomology (Figure S6D).

## Discussion

The constitutional 22q13 deletion is a fairly recently described genomic disorder that results in global developmental delay, delayed/absent speech, hypotonia and minor dysmorphic features. In spite of the fact that to date more than 100 cases (excluding ring 22s) have been detected by different molecular methods, when and how terminal deletions arise is still poorly understood. We have characterized from the clinical and molecular points of view 44 subjects with PMS resulting from simple 22q13 deletions (30 subjects, 72%), ring chromosomes (six subjects, 14%), unbalanced translocations (three subjects, 7%) and interstitial deletions (five subjects, 9%); all rearrangements result in haploinsufficiency of the *SHANK3* gene (Table 1). We have also determined the breakpoint junction sequences of twenty subjects with terminal deletions, five with interstitial deletions, one with ring 22 and three with unbalanced translocations.

### Clinical profile and genotype/phenotype comparison

Although in our cases age at diagnosis ranged from birth to 41 years, no specific clinical phenotype diagnostic for 22q13 deletion could be identified at any age (Table S1), as already noted by Phelan et al. [13]. Thus, successful diagnosis of this syndrome depends almost exclusively on the use of molecular diagnostic tools, mainly subtelomeric FISH and high-resolution genome-wide array-CGH. The latter is also suitable to identify cryptic interstitial deletions involving only the *SHANK3* gene, that are associated with an even less specific phenotype, as observed in our five patients (P37, P38, P42–P44). Their phenotype consisted mainly of developmental and language delay. PMS-suggestive facial dysmorphisms and hypotonia (limited to abdominal muscles) were observed only in one patient (P37), while no other physical abnormalities were noted in any of the patients. (Table 2). In patient P43, a defect in the abdominal wall with gut protrusion was detected by ultrasound during pregnancy and surgically corrected immediately after birth.

Owing to its emerging role in neuropsychiatric disorders and to the phenotypic overlap between autism and PMS, *SHANK3* has become a target for mutation screening in patients with autistic spectrum disorders (ASD) and several studies [19]–[21] have discovered *de novo* mutations in such patients. Mutations in *SHANK3* have also been found in schizophrenia [22] and non-syndromic intellectual disability [23]. The contribution of additional genes to the 22q13 deletion phenotype has also been debated. Very recently it has been proposed that deletion of the *IB2* gene (also named *MAPK8IP2* or *JP2*), mapping 70 kb proximal to *SHANK3*, may play a relevant role in PMS-associated ASD [24].

Two of our patients with interstitial microdeletions disrupting *SHANK3* and *ACR* only (P38, P43) (Figure 3B) fulfill the clinical criteria for a diagnosis of autism, while the others (P37, P42, P44), do not (Table 2). Our findings emphasize the incomplete penetrance of the ASD phenotype in PMS, while confirming a role for *SHANK3* in ASD. Additional deleted genes may contribute more strongly to accessory features, such as dysmorphisms and hypotonia, than to developmental and language delay.

Longitudinal clinical data on adult patients were collected in three subjects aged 40, 41 and 47 years. The severe progressive neurological deterioration reported in adult patients P10 (starting when she was 39 years old) and P30 (aged 40 years) was also described by Anderlid in a 30-year-old patient [25]. The minimal overlapping 22q13 region deleted in these three cases contains only *SHANK3*, *RABL2B* and *ACR*. In addition, subject P37 carrying an interstitial deletion involving only *SHANK3* experienced tremors and tics starting at age 23 (Text S1). Shank proteins, that organize glutamate receptors at excitatory synapses, are dramatically altered in Alzheimer disease [26]. In turn, disruption of glutamate receptors at the postsynaptic platform had been reported to contribute to the destruction of the postsynaptic density underlying mental deterioration in Alzheimer disease [27]. According to our results, *SHANK3* defects might indeed be responsible for progressive neurodegeneration, in addition to causing the neurobehavioral phenotype of the 22q13 syndrome.

Previous studies on a large cohort of patients with ring 22 demonstrated that there is considerable molecular and phenotypic overlap between individuals with ring 22 and those with del 22q13 [28]–[29]. All six subjects reported here showed features commonly found in 22q13.3 deletion syndrome, including accelerated growth in two of them (P26, P27), whereas one (P25) had slightly delayed growth.

### Parental origin

Parental origin was determined in 30/44 cases. We observed a larger proportion of 22q13 deletions of paternal (22/30, 73%), compared to maternal (8/30, 27%) origin, in agreement with a previous large study in which 69% of the deletions were of paternal origin [14]. There was no deletion size bias. Interestingly, we observed that both recurrent deletions (P31, P32), as all previously reported cases [18], [19], [30], were of paternal origin. Furthermore, the two interstitial deletions (P37, P38) we characterized were also paternal. In other terminal deletion cohorts, the majority of patients carry small 1p36 deletions on the maternal chromosome, while larger deletions are predominantly paternal [31]. In contrast, *de novo* simple small terminal 9q34.3 deletions are predominantly paternal, whereas larger terminal deletions, interstitial deletions, complex rearrangements and unbalanced translocations are frequently maternal in origin [12].

### Telomere healing and capture in terminal deletions

Broken chromosome ends can be stabilized through at least three mechanisms: *de novo* telomere addition mediated by telomerase; telomere capture resulting in a derivative chromosome; stabilization by break-fusion-break (BFB) cycles, generating terminal deletions and proximal inverted duplications. The first two mechanisms have been identified in this study. Almost 60% of our patients carried apparently simple terminal deletions. Nineteen of the twenty-two breakpoints we cloned, including all three breakpoints in mosaic subject P20, show evidence of telomere healing. Fourteen breakpoints contain 1–5 base microhomologies with the canonical GGTTAG sequence at the fusion point of genomic and telomeric sequences (Figure S2), possibly reflecting the template-driven mechanism that telomerase uses to replicate chromosome ends [32], [33]. The same mechanism applies to terminal 4p deletions [34] where microhomology with telomere repeats was found in all analyzed subjects. Human telomeres contain large blocks of 100–300 kb TAR sequences, located just proximally to the (TTAGGG)n tandem repeats, providing significant sequence homology between non-homologous chromosome ends [35]. Two terminal deletions in our cohort (P2, P39) were healed by telomere capture of TAR sequences (Figure S2). One deletion (P40) was repaired by the capture of the distal portion of Xp/Yp (Figure S2).

Failure to identify the nine remaining breakpoints may be due to the presence of regions containing large repetitive sequences or other complex sequences that would hinder amplification. Alternatively, some of the deletions may lack a telomere repeat at the breakpoint because the deletion may have been repaired by alternative mechanisms.

The presence of short repetitive elements may play a role in generating or stabilizing terminal deletions. In this study, we have determined 42 breakpoint junctions within the 22q13 region. Repetitive sequences, such as *Alu*, LINE, SINE, LTR and simple repeats were often, but not always, present at or near the breakpoints (Table S2). These repetitive elements are susceptible to DSBs due to replication errors or to the formation of unusual secondary structures, including cruciforms, hairpins, and tetraplexes [36]. On the other hand, there is no hard proof that the breakpoints of terminal deletions are the actual site of the original DSB, rather than the site where telomerase was able to synthesize a new telomere sequence.

Analysis of case P20 revealed a mosaic of at least three cell lines carrying different terminal 22q13 deletions. Their breakpoints were located approximately 100 Kb from each other. This mosaicism may be due to distinct stabilizing events, occurring in different cells of the early embryo, of the same unstable terminal deletion. Our results demonstrate that primary terminal deletion breakpoints and repair sites are not necessarily coincident and can actually be far apart. We had already shown, in an exceptional case of mosaicism for maternal 22q13.2-qter deletion (45% of cells) and 22q13.2-qter paternal segmental isodisomy (55% of cells) that complex mosaicism can also arise from a postzygotic or early embryonic recombination event [16]. These data suggest that terminal deletions can be repaired by multistep healing events. Cryptic mosaics may also render genotype-phenotype relationship in deletions more complex than expected.

Deletion sizes in patients with monosomy 1p36 [31] and 9p21–p24 [37] vary widely, up to 20 Mb, while 9q34.3 deletions [12] do not exceed 3.5–4 Mb. The size of 22q13 deletions is highly variable, ranging from 100 kb to 9 Mb [14]. No single common breakpoint has been discovered in deletions of 1p36 [31] and 9q34.3 [12], both studied in great detail. In contrast, 9 cases with terminal 140 kb deletion and a breakpoint occurring in a short GC-rich simple repeat in intron 8 of the *SHANK3* gene have been reported [18], [19], [25], [30], [38], [39]. In this study, we detected two new unrelated cases (P31, P32) with the same recurrent terminal deletion healed by *de novo* telomere addition. Computational analysis [40] predicts that this repeat would be able to form a secondary structure that may predispose to DNA double strand breaks, stabilize the broken chromosome end, or recruit telomerase more efficiently [36]. Subject P39 has a slightly larger deletion repaired by the capture of a TAR sequence.

### Interstitial deletions

Interstitial deletions affecting the 22q13 region have previously been described in three cases, one disrupting the *SHANK3* gene [41] and two more proximal [42]; none of them has been finely characterized at the molecular level. We characterized five additional *de novo* interstitial deletions between 17 and 74 Kb in size and sequenced their breakpoints: three of the deletions (P37, P44) disrupt exclusively *SHANK3*, the others (P38, P42, P43) both *SHANK3* and *ACR* (Figure 3B).

The interstitial deletions in four patients (P37,38, P42, P44) are compatible with NHEJ repair. P42 carries a more complex rearrangement where 40–47 bp from the deleted region are inserted in opposite orientation in the middle of the breakpoint junction (Figure 3C). A DNA replication model named FoSTeS [43], later generalized to the microhomology-mediated break-replication (MMBIR) model [44], has been proposed to explain complex rearrangements associated with several diseases. The rearrangement in P42 can indeed be explained by the FoSTeS/MMBIR mechanism (Figure 3D).

Apart from the cases described in this report, we have no information on the percentage of defects in *SHANK3* caused by deletions/duplications involving only one or a few exons. The small size of these rearrangements poses substantial problems for their identification, at least with current commercial aCGH platforms having necessarily limited coverage of the *SHANK3* gene. Arrays designed for the detection of clinically relevant exonic CNVs [45] may offer a solution.

### Ring 22 chromosomes and unbalanced translocations

NHEJ is the most likely repair mechanism leading to ring 22 formation in case P26. As this is the only ring 22 breakpoint we were able to clone, we cannot be sure that the same mechanism will apply to all cases with ring 22. Our inability to capture more breakpoints of ring 22 deletions may stem from the occurrence of the 22p breakpoints within highly repetitive sequences. Generation of breakpoints at both arms of the same chromosome, followed by circularization, has been usually assumed to be the basis of ring chromosome formation. Alternatively, telomere healing through circularization after the occurrence of a simple distal deletion, as it seems the be the case for ring chromosomes with concurrent deletion and duplication at one end [10], cannot be excluded. Thus, distal deletions and ring chromosomes might share the same initial event.

We also demonstrated that the complex phenotype in one ring 22 patient (P28) can be explained by the presence of further chromosome duplications at 22q11–12.3 and 22q12.3–13.2, undetectable with conventional cytogenetic analysis, in addition to the 22q13.3 deletion. The identification of the complex ring 22 rearrangement in this patient directly stems from the whole-genome aCGH analysis required by our protocol in order to exclude additional genomic aberrations.

All unbalanced translocations we analyzed (P11, P15, P16) were inherited from a parent carrying a balanced translocation. The microhomology found at all breakpoints points to NHEJ or MMBIR as the most likely mechanisms for these rearrangements; therefore they should be considered mechanistically different from all previously discussed chromosome 22 rearrangements.

### Conclusions

All adult patients with 22q13 deletion showed progressive clinical deterioration, supporting the hypothesis of a role for *SHANK3* haploinsufficiency in neurological deterioration. All patients with interstitial deletions involving only *SHANK3* showed a neurological and behavioral phenotype, demonstrating once again the specific role of the gene in this syndrome.

The study of breakpoints in subjects with 22q13 deletion provides a realistic snapshot of the variety of mechanisms driving non-recurrent deletion and repair at chromosome ends, including *de novo* telomere synthesis, telomere capture and circularization. Distinct stabilizing events of the same terminal deletion can also occur in different early embryonic cells. These data are in agreement with those demonstrating that mosaic structural chromosome abnormalities are common in early IVF embryos [46] and that chromosomally unbalanced zygotes are submitted, during first mitotic divisions, to intense genomic reshuffling eventually leading to different situations, all compatible with survival [47]. As recently suggested, the burst of DNA replication that accompanies the rapid cell division required to go from a single post-zygotic cell to an embryo and then a fetus is a time in the human life cycle when more new mutations may occur than was previously appreciated. Depending on the timing, many such events may be difficult, if not impossible, to identify at the DNA level [48].

## Materials and Methods

### Ethics statement

The study was approved by the Ethical Committee at the “Eugenio Medea” Scientific Institute.

### Human subjects

Blood samples were obtained from probands and their parents after informed consent. All patients were referred for genetic evaluation to different medical centers because of developmental delay, delayed/absent language and dysmorphic features. Physical examination and review of medical and family history records were performed on each patient. The diagnosis of terminal 22q13 deletion syndrome had not been proposed in any of the patients before identification of the deletion by cytogenetic or molecular diagnostic analysis. Cytogenetic and molecular diagnosis had been obtained by conventional karyotyping, subtelomere FISH, 22q13 MLPA analysis, or oligonucleotide-based aCGH (44k, 105k, 180k or 244k Agilent platforms)(Table 1).

### Array-CGH studies

A very high-resolution 22q13 custom array was designed using the eArray software (http://earray.chem.agilent.com/); probes were selected among those available in the Agilent database (UCSC hg18, http://genome.ucsc.edu). A total of 24624 probes were selected within the distal 9.4 Mb region of 22q13 (chr22: 40269203–49565875), and 8660 probes within the distal ∼3.2 Mb of chromosome 12q (chr12: 129000012–132289374); the latter set was used to identify the breakpoint interval in cases with a derivative chromosome 22 associated with a 12q genomic segment (P11, P15, P16), and for quality control/normalization. The probes provided an average resolution of 400 bp. Genomic DNA was isolated from blood samples using the GenElute-Blood kit (Sigma). Gender-matched genomic DNAs were obtained from individuals NA10851 (male) and NA15510 (female) (Coriell). The quality of each DNA was evaluated by conventional absorbance measurements (NanoDrop 1000, Thermo Scientific) and electrophoretic gel mobility assays. Quality of experiments was assessed using Feature Extraction QC Metric v10.1.1 (Agilent). The derivative log ratio spread (DLR) value was calculated using the Agilent Genomics Workbench software. Only experiments having a DLR spread value <0.30 were taken into consideration.

### Cytogenetic and FISH analysis

Metaphase chromosomes and interphase nuclei were obtained from all patients and their parents from PHA-stimulated blood lymphocyte cultures. G-banding karyotypes at 400–550 bands resolution were performed using standard high-resolution techniques. FISH experiments with 22q13.3 subtelomeric cosmids n66c4 (AC000050), n85a3 (AC000036), n94h12 (AC0020556) and n1g3 (AC002055) [18] were performed to confirm the aCGH results in cases where the 22q13.3 deletion disrupted the *SHANK3* gene (P31–32 P37–38, P42–43). FISH analysis with BAC clones, labeled with biotin-dUTP (Vector laboratories, Burligame, CA) using a nick translation kit (Roche), or probes for all subtelomeric regions (TelVysion kit, VYSIS) were performed on selected cases. The pan-telomeric peptide nucleic acid (PNA) probe (PNA FISH kit/Cy3, Dako Denmark A/S) which recognizes the consensus sequence (TTAGGG)n of human pan-telomeres was hybridized according to the manufacturer's instructions.

Hybridizations were analyzed with an Olympus BX61 epifluorescence microscope and images were captured with the Power Gene FISH System (PSI, Newcastle-upon-Tyne, UK).

### Parental origin determination

Genotyping of polymorphic sequence-tagged sites (STS) was performed by amplification with primers labeled with fluorescent probes followed by analysis on an ABI 310 Genetic Analyzer (Applied Biosystems). In cases where STS analysis was not informative, SNP genotyping was performed by PCR amplification followed by sequencing. All amplifications were performed with AmpliTaq Gold (Applied Biosystems) using standard protocols.

### Real-time PCR and MLPA analysis

Chromosome-specific target sequences for quantitative PCR analysis were selected within non- repeated sequences using Primer Express 2.0 software (Applied Biosystems) as described in Bonaglia et al. [18]. The annotated genomic sequence of chromosome 22 (March 2006 assembly, hg18) is available through the UCSC Human Genome Browser (http://genome.ucsc.edu/). Multiplex Ligation-dependent Probe Amplification analysis (MLPA) of the 22q13 region was performed with the SALSA MLPA kit P188 22q13 (MRC-Holland, Amsterdam).

### Breakpoint cloning

Amplification of 22q13 deletions repaired by chromosome healing was performed as in Bonaglia et al [18]. PCR products were both directly sequenced and cloned with a TOPO TA cloning kit (Invitrogen), followed by sequencing of individual clones. Inverse PCR was performed on *Sau3A*-cut, ligated (in 1 ml volume to facilitate self-ligation of individual fragments) genomic DNA, using nested sets of primers. Long-range PCRs were performed with JumpStart Red ACCUTaq LA DNA polymerase (Sigma) and the following protocol: 30 sec at 96°C, 35 cycles of 15 sec at 94°C/20 sec at 58°C/15 min at 68°C, 15 min final elongation time. Sequencing reactions were performed with a Big Dye Terminator Cycle Sequencing kit (Applied Biosystems) and run on an ABI Prism 3130xl Genetic Analyzer. Primer sequences are available in Table S2.

### Web resources

The accession number and URLs for data presented herein are as follow: UCSC Human Genome Browser, http://genome.ucsc.edu/; Online Mendelian Inheritance in Man (OMIM), http://www.ncbi.nlm.nih.gov/omim.

## Supporting Information

Figure S1 Photographs of adult patients. Top, Subject P30 at the age of 9 months (A), 13 months (B), 4 years (C); 8 years (D) and 35 years (E). No significant craniofacial dysmorphisms can be noticed, except for pointed chin (A,B,D), wide nasal bridge (A,C), bulbous nose (C,D,E). Middle, Subject P10 at the age of 12 years (I) and at the age of 40 years; frontal (G,H) and lateral (I) views. Note long face, large ears, full brow, prominent nasal bridge, long and bulbous nose, short philtrum, asymmetric mouth, thick lips. Bottom, Subject P33 in infancy (K), adolescence (L) and frontal (M) and lateral (N) views at the age of 41 years. Note the long eyelashes, full eyebrows, long and prominent nose, low forehead, micrognathia, thick hair, large ears, face asymmetry with hypo-mobility of the left side, small mandible.
(TIF)

Figure S2 Sequences of all breakpoint junctions of terminal and interstitial 22q13.3 deletions. The location of all sequences on the hg18 Human Genome sequence is indicated. Repetitive sequences are shown in lowercase letters. The identity of all repetitive sequences is indicated in the Repeats column. Telomere repeat sequences are shown in red. Genomic sequences with microhomology to the telomere repeat are underlined. TAR sequences are shown in blue. Microhomologies at the junctions of interstitial deletions are shown in bold.
(PDF)

Figure S3 Molecular characterisation of the 22q13.2 terminal deletion in subject P12. A, Whole chromosome view and B, detail of array-CGH analysis using an oligonucleotide-based custom 22q13 microarray. Arrowheads delimit two mosaic deleted regions: the BP1–BP2 deletion region (from 44,606 kb to 45,600 kb) has an average log ratio of −0.8; the deleted region between BP2 and the telomere (from 45,600 to 49,566 kb) has an average log ratio of −1.0. C, Tel-ACP amplification and direct sequencing of the amplified fragments revealed the breakpoint junction at BP1. A telomere repeat is present at the breakpoint. Repetitive sequences are shown in lowercase letters. Telomere repeat sequences are shown in red. Genomic sequences with microhomology to the telomere repeat are underlined.
(PDF)

Figure S4 Molecular characterisation of ring chromosome 22 in subject P28. Whole chromosome 22 view (left) and details (right) of a 180k Agilent array-CGH profile showing the 18 Mb duplication at 22q11–12.3, the 4.2 Mb duplication at 22q12.3–13.2 and the distal 240 kb deletion at 22q13.3.
(PDF)

Figure S5 Molecular characterisation of the ring 22-associated deletion in subject P26. A, Whole chromosome view (left) and detail (right) of array-CGH analysis using a 180k Agilent kit microarray. B, Inverse-PCR amplification and direct sequencing of the amplified fragments revealed the breakpoint junction. Repetitive sequences are shown in lowercase letters. C, FISH analysis using the PAN-Tel probe confirmed the deletion of the 22p and 22q telomeres of ring chromosome 22 (arrow).
(PDF)

Figure S6 Molecular characterisation of the 22q13.2 terminal translocations in subjects P11, P15/P16. A, details of the of array-CGH analysis using an oligonucleotide-based a 44k Agilent kit microarray showing the breakpoint regions on chromosome 22q (left) and 12q (right) in case P11. B, Long-range PCR amplification and direct sequencing of the breakpoint junction. Repetitive sequences are shown in lowercase letters. Microhomologies at the junction are shown in bold. C, details of the of array-CGH analysis using an oligonucleotide-based 22q13 custom array (eArray, Agilent) showing the breakpoint regions on chromosome 22q (left) and 12q (right) in cases P15/P16. D, Long-range PCR amplification and direct sequencing of the breakpoint junction.
(JPG)

Table S1 Clinical features of PMS patients compared to the subjects in this study. (*) Prevalence according to Phelan, 2007 (Ref. [17]). (a) Accelerated growth was observed in 9 cases out of 29, including the two patients (P26, P27) with ring 22 for whom this information was available. In one patient (P25) with ring 22, growth was slightly delayed, while short stature (<3^rd^ centile) was observed in one subject (P38). (b) Brain imaging studies, performed in 23 subjects, showed abnormal focal signals in 5 patients (22%), diffuse hyperintensities of white matter in three (13%), thin or short corpus callosum in 4 (17,4%), asymmetry or enlargement of lateral ventricles in 6 (26%) and arachnoid cysts in two patients (8.6%); the remaining three cases had normal brain MRI. (c) According to Havens et al. 2004 (Havens JM, Visootsak J, Phelan MC, Graham JM Jr (2004) 22q13 deletion syndrome: an update and review for the primary pediatrician. Clin Pediatr (Phila) 43: 43–53.) (d) Renal problems, including hydronephrosis (P5, P36), right renal agenesis (P10), hypoplasia of right kidney (P27) were ascertained in 10% of cases. (e) The following behavioral disturbances were observed: hyperactivity, stereotypes, poor concentration, poor social interactions, poor eye contact, excessive screaming, and aggressiveness.
(PDF)

Table S2 Primers used for breakpoint cloning. Primer names, sequences and amplification methods are indicated. Nested PCR primers are indicated as F2, R2, F3, R3.
(PDF)

Text S1 Supplementary Clinical Information.
(PDF)
