# Supplementary figures and images for: Molecular Mechanisms Generating and Stabilizing Terminal 22q13 Deletions in 44 Subjects with Phelan/McDermid Syndrome
Source: PLoS Genet. 2011 Jul 14;7(7):e1002173. doi: 10.1371/journal.pgen.1002173 (PMC3136441; doi:10.1371/journal.pgen.1002173)

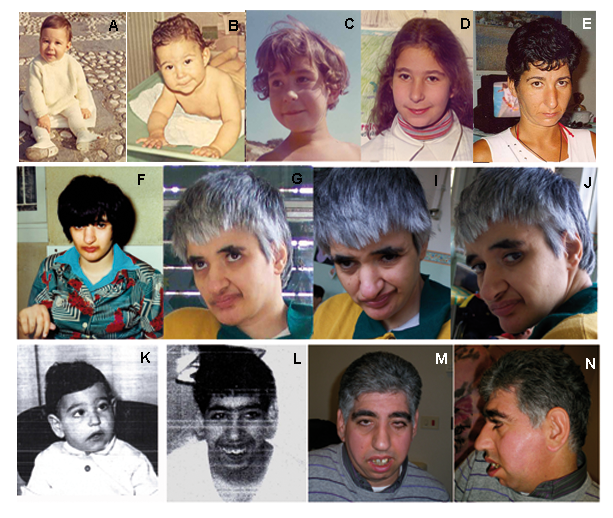

Supplement: Figure S1 — Photographs of adult patients. Top, Subject P30 at the age of 9 months (A), 13 months (B), 4 years (C); 8 years (D) and 35 years (E). No significant craniofacial dysmorphisms can be noticed, except for pointed chin (A,B,D), wide nasal bridge (A,C), bulbous nose (C,D,E). Middle, Subject P10 at the age of 12 years (I) and at the age of 40 years; frontal (G,H) and lateral (I) views. Note long face, large ears, full brow, prominent nasal bridge, long and bulbous nose, short philtrum, asymmetric mouth, thick lips. Bottom, Subject P33 in infancy (K), adolescence (L) and frontal (M) and lateral (N) views at the age of 41 years. Note the long eyelashes, full eyebrows, long and prominent nose, low forehead, micrognathia, thick hair, large ears, face asymmetry with hypo-mobility of the left side, small mandible. (TIF) [file pgen.1002173.s001.tif]

# Subject P28

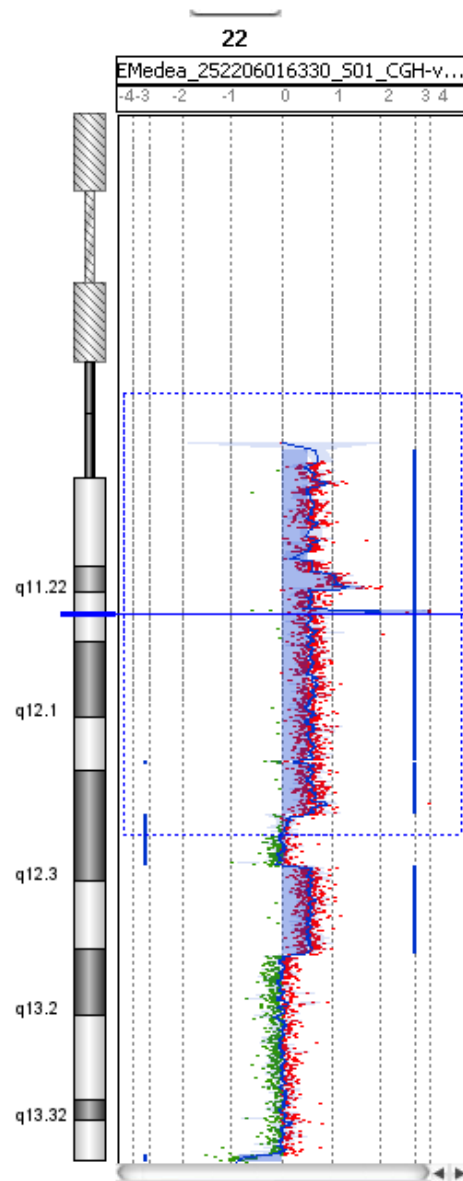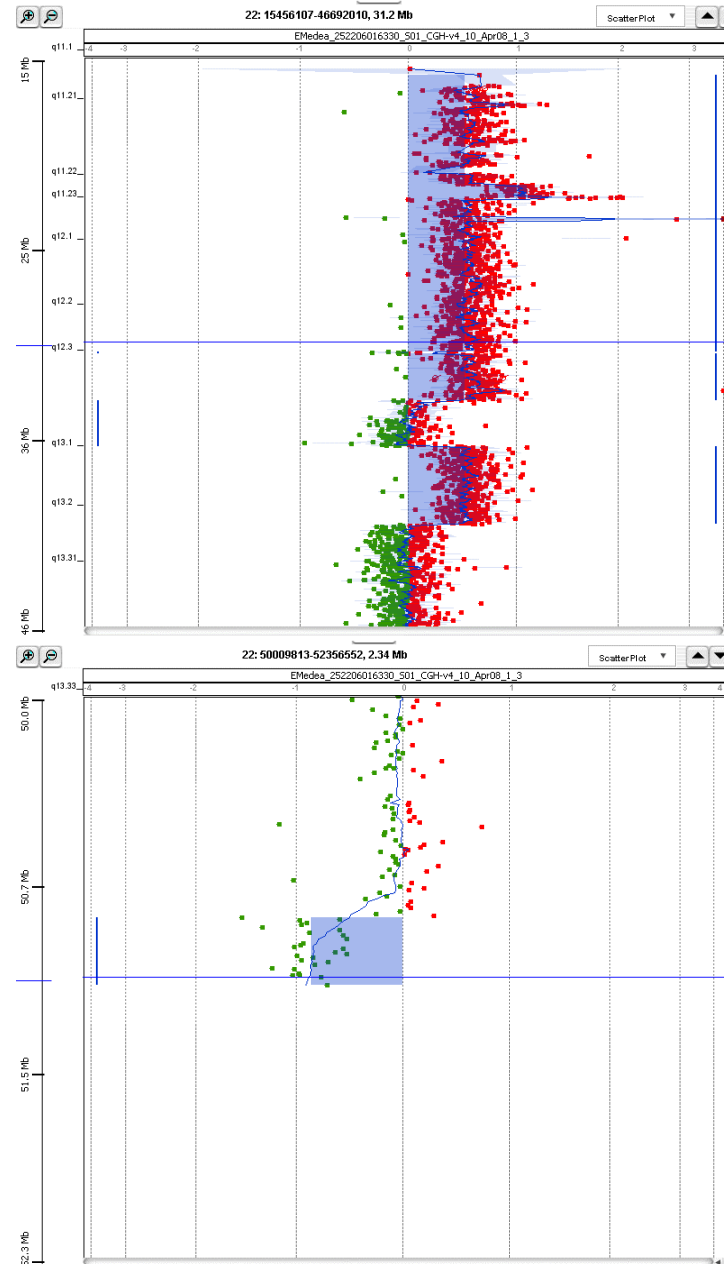

**22q11-12.3 duplication**  
**size: 18 Mb**

**22q12.3-13.2 duplication**  
**size: 4.2 Mb**

**22q13.3 deletion**  
**Size: 240 kb**

Supplement: Figure S4 — Molecular characterisation of ring chromosome 22 in subject P28. Whole chromosome 22 view (left) and details (right) of a 180k Agilent array-CGH profile showing the 18 Mb duplication at 22q11–12.3, the 4.2 Mb duplication at 22q12.3–13.2 and the distal 240 kb deletion at 22q13.3. (PDF) [file pgen.1002173.s004.pdf]

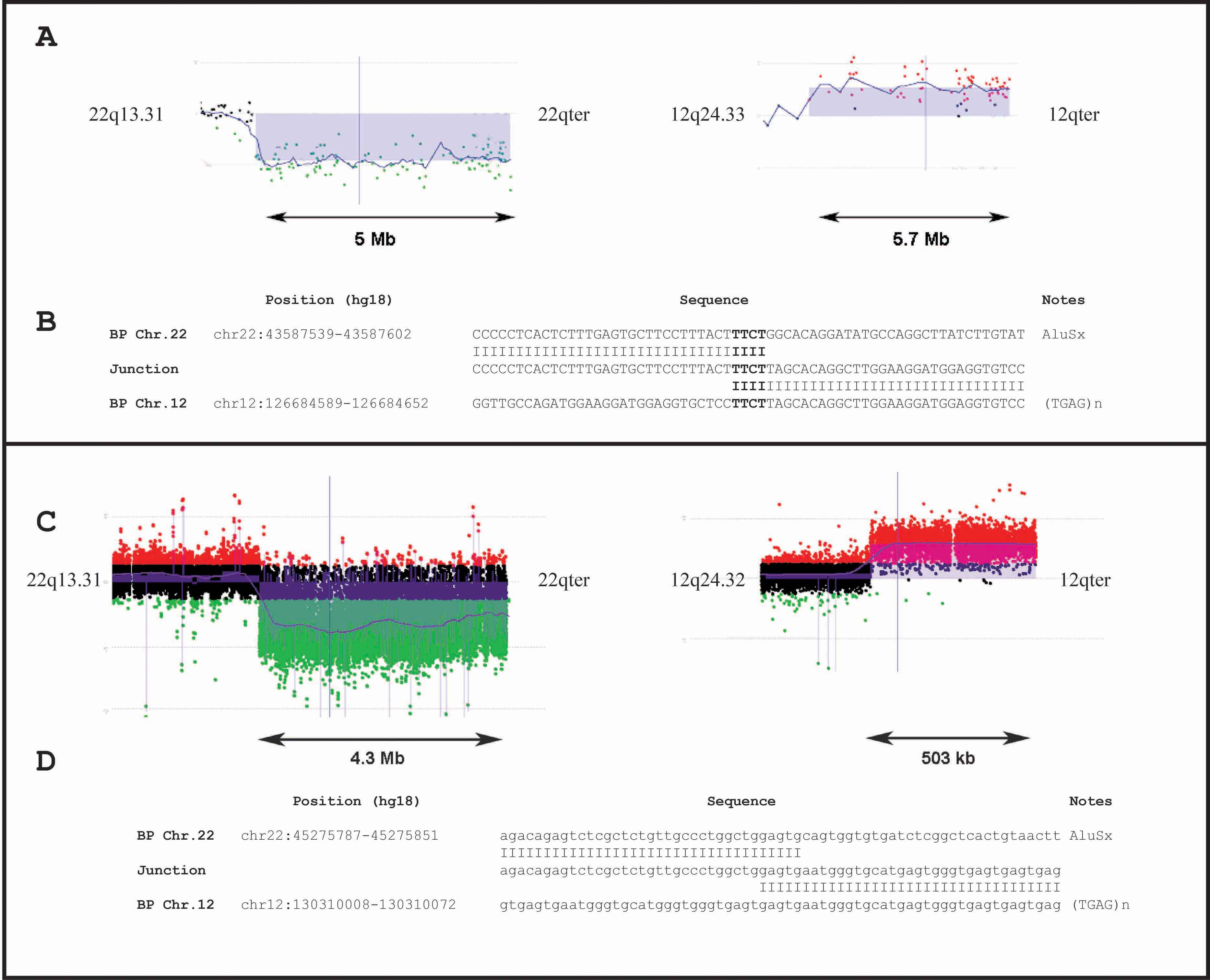

Supplement: Figure S6 — Molecular characterisation of the 22q13.2 terminal translocations in subjects P11, P15/P16. A, details of the of array-CGH analysis using an oligonucleotide-based a 44k Agilent kit microarray showing the breakpoint regions on chromosome 22q (left) and 12q (right) in case P11. B, Long-range PCR amplification and direct sequencing of the breakpoint junction. Repetitive sequences are shown in lowercase letters. Microhomologies at the junction are shown in bold. C, details of the of array-CGH analysis using an oligonucleotide-based 22q13 custom array (eArray, Agilent) showing the breakpoint regions on chromosome 22q (left) and 12q (right) in cases P15/P16. D, Long-range PCR amplification and direct sequencing of the breakpoint junction. (JPG) [file pgen.1002173.s006.jpg]
