# Supplementary material for: Molecular Mechanisms Generating and Stabilizing Terminal 22q13 Deletions in 44 Subjects with Phelan/McDermid Syndrome
Source: PLoS Genet. 2011 Jul 14;7(7):e1002173. doi: 10.1371/journal.pgen.1002173 (PMC3136441; doi:10.1371/journal.pgen.1002173)
Supplement: Figure S2 — Sequences of all breakpoint junctions of terminal and interstitial 22q13.3 deletions. The location of all sequences on the hg18 Human Genome sequence is indicated. Repetitive sequences are shown in lowercase letters. The identity of all repetitive sequences is indicated in the Repeats column. Telomere repeat sequences are shown in red. Genomic sequences with microhomology to the telomere repeat are underlined. TAR sequences are shown in blue. Microhomologies at the junctions of interstitial deletions are shown in bold. (PDF) [file pgen.1002173.s002.pdf]

Terminal deletions

P01	Position			Repeats
BP Chr. 22	chr22:48609895-48609954	ggccgaggcgggcagactacctgaggtcgg	gagttcaagaccagcctgatcaacatggag	AluSp
Junction		ggccgaggcgggcagactacctgaggtcgg	TTAG(GGTTAG)n	

P02	Position	Sequence		Repeats
BP Chr. 22	chr22:44280750-44280809	GGGAAGAGCGCAATTCTGCCAATACCCCAG	AATTGTTTTCCCCAAGATGCCTCTGCACAT	None
Junction		GGGAAGAGCGCAATTCTGCCAATACCCCAG	GGAGAG(GGTGAG)n	

P03	Position	Sequence		Repeats
BP Chr. 22	chr22:47183281-47183340	tgtaatcccagcactttgggaggccaaggt	gggtggataattggaggtcaggagtttgag	AluSx
Junction		tgtaatcccagcactttgggaggccaaggt	TAG(GGTTAG)n	

P04	Position	Sequence		Repeats
BP Chr. 22	chr22:48040155-48040214	GGCTGGCTCAGGCCGGGACTCTGGGTCAGG	TGCAGCCTCATGGAAAGACCTCTACAGGAG	None
Junction		GGCTGGCTCAGGCCGGGACTCTGGGTCAGG	GTTAG(GGTTAG)n	

P05	Position	Sequence		Repeats
BP Chr. 22	chr22:43136766-43136825	gttacagccaggggttagttatagccaggg	gttagttacagccaggggttagttatagcc	Simple tandem repeat
Junction		gttacagccaggggttagttatagccaggg	TTAG(GGTTAG)n	

P06	Position	Sequence		Repeats
BP Chr. 22	chr22:47040707-47040766	GGTAACCCAGCTGAGGTCCACAGATCACGG	ACAGGCATGCTGGGAGTGGCCACAGGGAGA	None
Junction		GGTAACCCAGCTGAGGTCCACAGATCACGG	TTAG(GGTTAG)n	

P07	Position	Sequence			Repeats
BP Chr. 22	chr22:46600236-46600295	AGGATGTTTTACAATACCAAGGGTGGAAAT		CCAACCCAACTCCCCTTTTGTAAAACAAAG	None
Junction		AGGATGTTTTACAATACCAAGGGTGGAAAT	ATCTTTGTGAGAACAGACAA	TAG(GGTTAG)n	

P08	Position	Sequence			Repeats
BP Chr. 22	chr22:41517849-41517908	CTGGGCCTCAGGAACTCTGCTGCTGCTGTT		GGGGCTGCCCCAAGCTAGCGTGGACAGAAG	None
Junction		CTGGGCCTCAGGAACTCTGCTGCTGCTGTT	TAACCCTAACTGCT	(GGTTAG)n	

P12	Position	Sequence		Repeats
BP Chr. 22	chr22:44623743-44623802	aatggagagagagacttgagttggaactgt	gtgtctgagggaggatccgtaggacaagct	L2
Junction		aatggagagagagacttgagttggaactgt	TAG(GGTTAG)n	

P13	Position	Sequence		Repeats
BP Chr. 22	chr22:48476926-48476985	actctagagtgaaggtaaaggcagttttag	accaagatcaaaagtttactatcagtgaaa	LIMEd
Junction		actctagagtgaaggtaaaggcagttttag	(GGTTAG)n	

P14	Position	Sequence		Repeats
**BP Chr. 22**	chr22:43867958-43868017	aggtctggaaagtccttaggcatgtgcagt	cacctcttcatgctacctcgtgtgtcacat	LTR45C
**Junction**		aggtctggaaagtccttaggcatgtgcagt	TAG(GGTTAG)n	

P20 BP1	Position	Sequence		Repeats
**BP Chr. 22**	chr22:42406130-42406189	AAAATCTGAAAAGAATATAAGGTTCTCTTG	AGGGGTAAATTAAGTACTTTAGGGGATATA	None
**Junction**		AAAATCTGAAAAGAATATAAGGTTCTCTTG	GTTAG(GGTTAG)n	

P20 BP2	Position	Sequence		Repeats
**BP Chr. 22**	chr22:42603398-42603457	ACTCCTGCCAAGAcaggtctaggtcaagat	taattgcattagaatctctgcagaagagcc	MER5b
**Junction**		ACTCCTGCCAAGAcaggtctaggtcaagat	GGTTAG(GGTTAG)n	

P20 BP3	Position	Sequence		Repeats
**BP Chr. 22**	chr22:42728708-42728767	aagcttattttataagaaagtattgaagat	cttgtgcagtttgttgaataccgtacATGA	Tigger5
**Junction**		aagcttattttataagaaagtattgaagat	AT TTAG(GGTTAG)n	

P21	Position	Sequence		Repeats
**BP Chr. 22**	chr22:44960699-44960758	ACTTAGATACCACAGGATTGGCAGTGTAGA	CTTACATTCATAGACCGGATGCCATCAGCC	None
**Junction**		ACTTAGATACCACAGGATTGGCAGTGTAGA	TTAG(GGTTAG)n	

P30	Position	Sequence		Repeats
**BP Chr. 22**	chr22:46018962-46019021	cttactattatgattttgttgtttatatgg	tgttttcttgctgtcatggtgaagtaatga	LIMCc
**Junction**		cttactattatgattttgttgtttatatgg	TAG(GGTTAG)n	

P31	Position	Sequence		Repeats
**BP Chr. 22**	chr22:49469041-49469100	Agagggggtggagaggggggtggtggaggg	gggtggtggagaggggggtggtggaggggg	G-rich, Low complexity (SHANK3-REP)
**Junction**		Agagggggtggagaggggggtggtggaggg	TTAG(GGTTAG)n	

P32	Position	Sequence		Repeats
**BP Chr. 22**	chr22:49469045-49469104	ggggtggagaggggggtggtggagggggtt	ggtggagaggggggtggtggagggggatgg	G-rich, Low complexity (SHANK3-REP)
**Junction**		ggggtggagaggggggtggtggagggggtt	AG(GGTTAG)n	

P34	Position	Sequence		Repeats
**BP Chr. 22**	chr22:45252070-45252129	attggctttttttttttttgagacggagt	cttgctctgtcaccaggctggagtgcagtg	AluSc
**Junction**		attggctttttttttttttgagacggagt	TAG(GGTTAG)n	

P36	Position	Sequence		Repeats
**BP Chr. 22**	chr22:40440085-40440144	caaccctttgaacaaatgagagattgaagt	ccagggaggaaaaggagctttcctgaggtc	MIR3
**Junction**		caaccctttgaacaaatgagagattgaagt	TAG(GGTTAG)n	

P39	Position	Sequence			Repeats
**BP Chr. 22**	chr22:49468904-49468963	GTCCCCTCGCCAGGGAGAGAGGAGGGTCAG		CAGGGCTCTGGGGCAGGGGTATGGGGAAAA	None
**Junction**		GTCCCCTCGCCAGGGAGAGAGGAGGGTCAG		$(GGTCAG)_6$(GGTTAG)$(GGTCAG)_2$(GGTTAG)n	

P40	Position	Sequence			Repeats
**BP Chr.22**	chr22:42119850-42119912	gcaaggtgatcagggtgggcttcttggagg	**agg**	tgatatctgaggtgagatctggaaagcaag	L2
**Junction**		gcaaggtgatcagggtgggcttcttggagg	**agg**	ggttgtctcagggtcctagtgtgtctggaa	
**BP Chr. X/Y**	chrX/Y:418-480 (rev.)	ggaactgggttaTCAACCAGGTGCTCTTCT	**AGG**	ggttgtctcagggtcctagtgtgtctggaa	LTR12d

Interstitial deletions

P37	Position (hg18)	Sequence			Repeats
BP1 Chr. 22	chr22:49417717-49417776	cacataataatgttgtggctagccttagaa		atcctcttcacaaaattaaagagcaaaaat	LTR49int
Junction		cacataataatgttgtggctagccttagaa	GCTGCAACCC	CAGGTGGTACGGGAGGGCAGGCGGTCCAGC	
BP2 Chr. 22	chr22:49491490-49491549	TCCATGATGGGCAGACAGGGCCGGGAGACA		CAGGTGGTACGGGAGGGCAGGCGGTCCAGC	None

P38	Position (hg18)	Sequence			Repeats
BP1 Chr. 22	chr22:49493608-49493667	cctcggcctcccaaagtgctgggattacag	**gcgt**	gagccactgcgcccagccAGAAGGATACAG	AluSg
Junction		cctcggcctcccaaagtgctgggattacag	**gcgt**	atgttgaaccagccttgcatcccagggatg	
BP2 Chr. 22	chr22:49537718-49537781	gtttatatactggattacatttatcagttt	**gcgt**	atgttgaaccagccttgcatcccagggatg	LIPA4

P42	Position (hg18)	Sequence	Repeats
**BP1 Chr. 22**	chr22:49511283-49511342	TCGGTAACCTGTGGAAAAATGATGAGCTCT**GGGG**CCACCTTTGATTTTCAGGGGAACCTG	None
**Junction**		TCGGTAACCTGTGGAAAAATGATGAGCTCT**GGGG**ACGGGCGGAGGGGAGCGGGCAGGG**TC**TCTACTGTGACCTGGGTGGGCGGTGCTC	
**BP2 Chr. 22**	chr22:49515909-49515996 (rev)	CAGCGCCAGGTGAGGGGCGCACAGCCAAGG**GGGG**ACGGGCGGAGGGGAGCGGGCAGGG**TC**CAGGAGGAAACGGGCTGGGGCTGCGGCT	None
**BP3 Chr. 22**	chr22:49528909-49528968	GTCCTGAGGCCAGGCTGCAGCCCCACCACC**TC**TCTACTGTGACCTGGGTGGGCGGTGCTC	None

P43	Position (hg18)	Sequence			Repeats
**BP1 Chr. 22**	chr22:49498120-49498182	GGCTGGTTGATCATCCCGCAGGCTGGGGCC	**CTG**	TTGATCAAACAGCTGCAAACCCAGCTGTGT	None
**Junction**		GGCTGGTTGATCATCCCGCAGGCTGGGGCC	**CTG**	CAAGAGAGATATGTGGAGAAAATCATCATT	
**BP2 Chr. 22**	chr22:49525034-49525096	TATGGGAACAATAAACCAGTAAAGGCGCCT	**CTG**	CAAGAGAGATATGTGGAGAAAATCATCATT	None

P44	Position (hg18)	Sequence		Repeats
**BP1 Chr. 22**	chr22:49433639-49433698	ttcaggcaattctcctgcctcagcctccca	agtagctgggattacaggcatgcaccacca	AluSx
**Junction**		ttcaggcaattctcctgcctcagcctccca	GAAGTGTGTGTTGAGGAAGTAACTGGGCCA	
**BP2 Chr. 22**	chr22:49472587-49472646	TGCCACAGCTGCTCTCAAGGCCACTGCAGG	GAAGTGTGTGTTGAGGAAGTAACTGGGCCA	None
