# Supplementary material for: Molecular Mechanisms Generating and Stabilizing Terminal 22q13 Deletions in 44 Subjects with Phelan/McDermid Syndrome
Source: PLoS Genet. 2011 Jul 14;7(7):e1002173. doi: 10.1371/journal.pgen.1002173 (PMC3136441; doi:10.1371/journal.pgen.1002173)
Supplement: Figure S3 — Molecular characterisation of the 22q13.2 terminal deletion in subject P12. A, Whole chromosome view and B, detail of array-CGH analysis using an oligonucleotide-based custom 22q13 microarray. Arrowheads delimit two mosaic deleted regions: the BP1–BP2 deletion region (from 44,606 kb to 45,600 kb) has an average log ratio of −0.8; the deleted region between BP2 and the telomere (from 45,600 to 49,566 kb) has an average log ratio of −1.0. C, Tel-ACP amplification and direct sequencing of the amplified fragments revealed the breakpoint junction at BP1. A telomere repeat is present at the breakpoint. Repetitive sequences are shown in lowercase letters. Telomere repeat sequences are shown in red. Genomic sequences with microhomology to the telomere repeat are underlined. (PDF) [file pgen.1002173.s003.pdf]

Subject P12

A

B

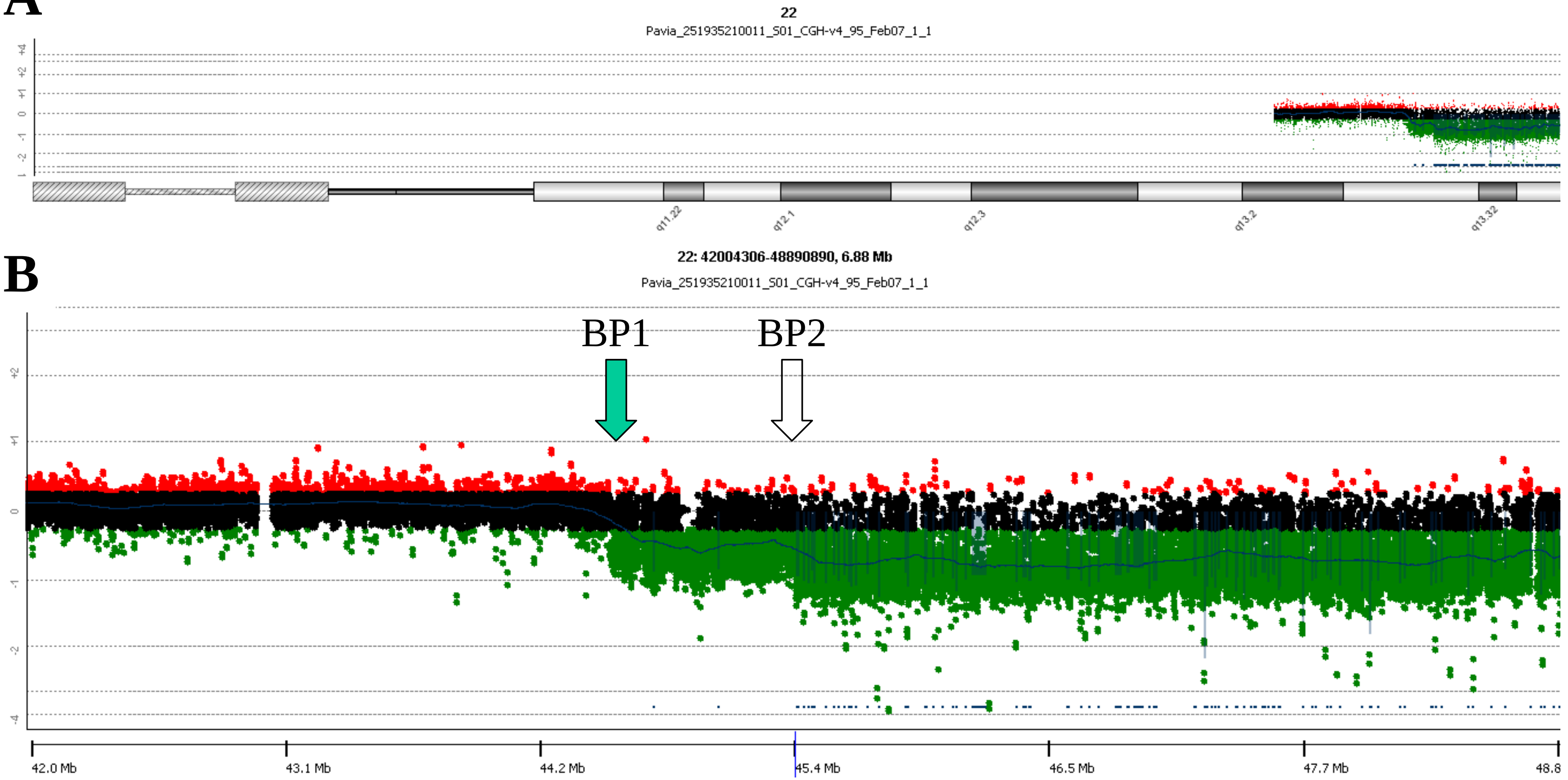

C

	Position	Sequence	
**BP1**	chr22:44623743-44623802	aatggagagagagacttgagttggaactgt	gtgtctgagggaggatccgtaggacaagct
		IIIIIIIIIIIIIIIIIIIIIIIIIIIIII	
**Junction**		aatggagagagagacttgagttggaact<u>gt</u>	TAG(GGTTAG)n
