# Supplementary material for: Molecular Mechanisms Generating and Stabilizing Terminal 22q13 Deletions in 44 Subjects with Phelan/McDermid Syndrome
Source: PLoS Genet. 2011 Jul 14;7(7):e1002173. doi: 10.1371/journal.pgen.1002173 (PMC3136441; doi:10.1371/journal.pgen.1002173)
Supplement: Figure S5 — Molecular characterisation of the ring 22-associated deletion in subject P26. A, Whole chromosome view (left) and detail (right) of array-CGH analysis using a 180k Agilent kit microarray. B, Inverse-PCR amplification and direct sequencing of the amplified fragments revealed the breakpoint junction. Repetitive sequences are shown in lowercase letters. C, FISH analysis using the PAN-Tel probe confirmed the deletion of the 22p and 22q telomeres of ring chromosome 22 (arrow). (PDF) [file pgen.1002173.s005.pdf]

Subject P26

A

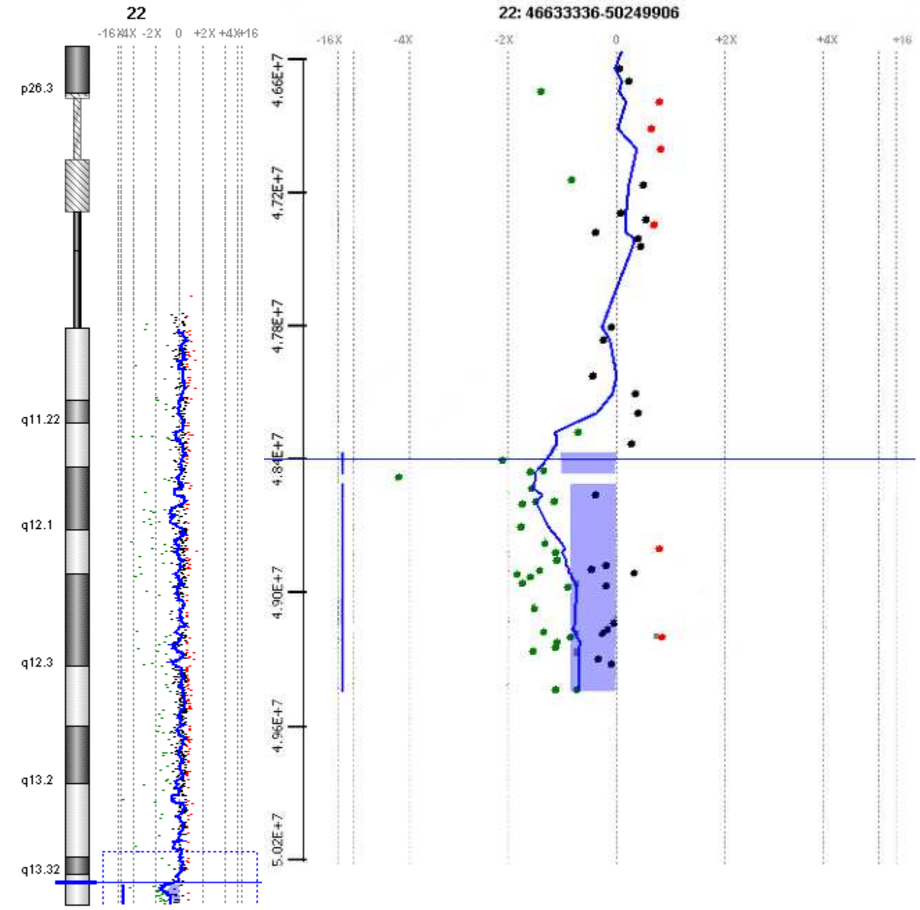

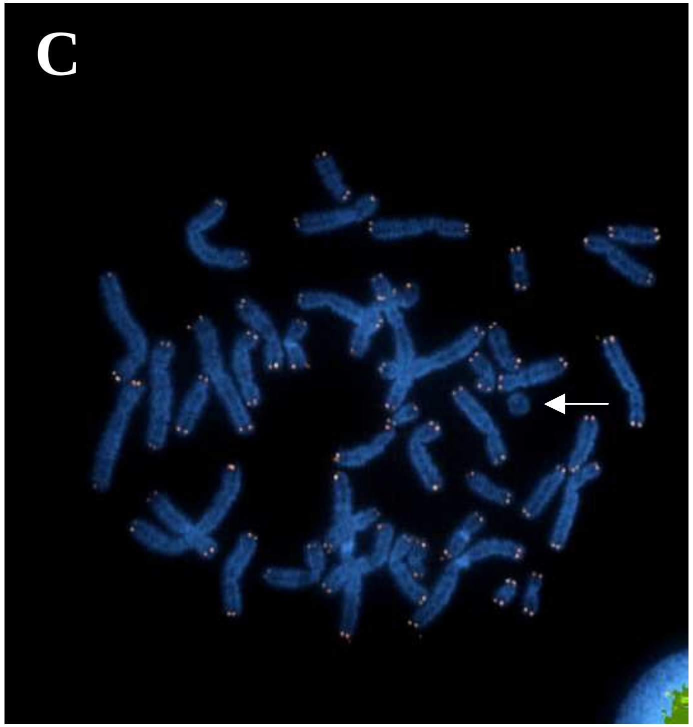

B

Chromosome	Position (hg18)	Sequence	
BP Chr. 22q	chr22:48448979-48449038	agagtgagcagcagcaagatttagcgcaaa	gagcaaaagaacaaagcttccacactgctg
		IIIIIIIIIIIIIIIIIIIIIIIIIIIIII	
Junction		agagtgagcagcagcaagatttagcgcaaa	ttcaatggaattcaatggaatgcaatggaa
			IIIIIIIIIIIIIIIIIIIIIIIIIIIIII
BP Chr. 22p	Many telomeres/centromeres		ttcaatggaattcaatggaatgcaatggaa
