# Supplementary material for: Molecular Mechanisms Generating and Stabilizing Terminal 22q13 Deletions in 44 Subjects with Phelan/McDermid Syndrome
Source: PLoS Genet. 2011 Jul 14;7(7):e1002173. doi: 10.1371/journal.pgen.1002173 (PMC3136441; doi:10.1371/journal.pgen.1002173)
Supplement: Table S1 — Clinical features of PMS patients compared to the subjects in this study. (*) Prevalence according to Phelan, 2007 (Ref. [17]). (a) Accelerated growth was observed in 9 cases out of 29, including the two patients (P26, P27) with ring 22 for whom this information was available. In one patient (P25) with ring 22, growth was slightly delayed, while short stature (<3rd centile) was observed in one subject (P38). (b) Brain imaging studies, performed in 23 subjects, showed abnormal focal signals in 5 patients (22%), diffuse hyperintensities of white matter in three (13%), thin or short corpus callosum in 4 (17,4%), asymmetry or enlargement of lateral ventricles in 6 (26%) and arachnoid cysts in two patients (8.6%); the remaining three cases had normal brain MRI. (c) According to Havens et al. 2004 (Havens JM, Visootsak J, Phelan MC, Graham JM Jr (2004) 22q13 deletion syndrome: an update and review for the primary pediatrician. Clin Pediatr (Phila) 43: 43–53.) (d) Renal problems, including hydronephrosis (P5, P36), right renal agenesis (P10), hypoplasia of right kidney (P27) were ascertained in 10% of cases. (e) The following behavioral disturbances were observed: hyperactivity, stereotypes, poor concentration, poor social interactions, poor eye contact, excessive screaming, and aggressiveness. (PDF) [file pgen.1002173.s007.pdf]

Table S1. Clinical features of PMS patients compared to the subjects in this study

Features	PMS patients*	Our study
Mean age at diagnosis		11.2 years
Born at term		85%
Born pre-term		15%
Walking		Mean age 24 months
**Neurologic**		
Develomental delay	>95%	100%
Delayed/absent language	>95%	100%
Neonatal hypotonia	>95%	77% (still present, 41%)
**Growth**		
Normal		69%
Accelerated		31%[a]
**Seizures**		
Clinical epileptic seizures	>25%	30%
Abnormal EEG epileptic anomalies		27%
Abnormal EEG with focal or diffused slow abnormalities		32%
**Facial dysmorphisms**		
Long eyelashes	>75%	55%
Dolichocephaly	>50%	18%
Full brow	>50%	43%
Flat midface	>50%	33%
Ptosis	>50%	4%
Large/prominent ears	>50%	65%
Wide nasal bridge	>50%	50%
Puffy cheeks	>50%	57%
Pointed chin	>50%	43%
Bulbous nose	>50%	59%
Widely spaced teeth/malocclusion	>25%	38%
Epicanthal folds	>25%	48%
**Extremities**		
Large and fleshy hands	>75%	48%
Dysplastic toenails	>75%	23%
2/3rd toe syndactyly	>25%	44%
5th finger clinodactyly	>25%	15%
**Other features**		
Decreased sensitivity to pain	>50%	69%
Hypohydrosis	>50%	17%
High tolerance to pain		69% (9/13)
Brain abnormalities at MRI		69%[b]
Cardiac anomalies	6%[c]	6,9%
Renal problems	>25%[d]	10%
**Behavioural features**		
Autism (DMS-IV)		13%
Behavioural disturbances		77%[e]
