# Supplementary material for: Molecular Mechanisms Generating and Stabilizing Terminal 22q13 Deletions in 44 Subjects with Phelan/McDermid Syndrome
Source: PLoS Genet. 2011 Jul 14;7(7):e1002173. doi: 10.1371/journal.pgen.1002173 (PMC3136441; doi:10.1371/journal.pgen.1002173)
Supplement: Table S2 — Primers used for breakpoint cloning. Primer names, sequences and amplification methods are indicated. Nested PCR primers are indicated as F2, R2, F3, R3. (PDF) [file pgen.1002173.s008.pdf]

Subject	Method	Primer F	Sequence	Primer F2	Sequence	Primer F3	Sequence	Primer R	Sequence	Primer R2	Sequence	Primer R3
**P01**	ACP-Tel	P1-22-F1	GACAGTGGCCTCTCAGACAA	P1-22-F2	TGATGCTCAGAGGAGTGAAGA			ACP-Tel	TCACAGAAGTATGCCAAGCGAIIIIIAACCCTAACCCT	UNI-ACP	TCACAGAAGTATGCCAAGCGA	
**P02**	ACP-Tel	P2-22-F1	GAGCCGCCTGTCTGTCCT	P2-22-F2	CAGGGAGGACTTGGCTTGA			ACP-Tel		UNI-ACP		
**P03**	ACP-Tel	P3-22-F1	TTCACAGCGTAATCGCAAATG	P3-22-F2	GCAGTAAGGAAAGTAGTAGGT			ACP-Tel		UNI-ACP		
**P04**	ACP-Tel	P4-22-F1	TCCTGGAACTCGACATGTTCA	P4-22-F2	CAGAGGTCACTGTTTTAGACAA			ACP-Tel		UNI-ACP		
**P05**	ACP-Tel	P5-22-F1	GTGCCCAAATGTGTCATTGTC	P5-22-F2	TCCAACAACTTCCGAACAGCA			ACP-Tel		UNI-ACP		
**P06**	ACP-Tel	P6-22-F1	GAGATGGTAGCAGAGTCAAGA	P6-22-F2	AACTTTCCATCTGTCCCTGAG	P6-22-F3	GGCCTTCGGACGCATTATGA	ACP-Tel		UNI-ACP		UNI-ACP
**P07**	ACP-Tel	P7-TEL-1F	GCACCATATCATTTACGGAAAG	P7-TEL-2F	AGCCTACAAAGAAGTTCCATCA			ACP-Tel		UNI-ACP		
**P08**	ACP-Tel	P8-22-F1	GGAGGCTCAGTGCACATTCAA	P8-22-F2	GGGAAGTCCTCAGAACTCAG			ACP-Tel		UNI-ACP		
**P11**	LR-PCR	P11-22-F	AGTGCTTGCCTCACCAAAGA					P11-12-R	TCGGCAGCTCCTTTCTCATC			
**P12**	ACP-Tel	P12-22-F1	CGGTGCTGTCCAAGGTTCT	P12-22-F2	CTGTTTCCCTCTTTCCTACACA			ACP-Tel		UNI-ACP		
**P13**	ACP-Tel	P13-22-F1	TCCACAAATCAGGGCACAAGA	P13-22-F2	GAACCCACGGGATGTTCTCA	P13-22-F3	CAGGTTAGGCAGTGACTACTC	ACP-Tel		UNI-ACP		UNI-ACP
**P14**	ACP-Tel	P14-22-1F	ACCCAGTCTCTCTTGACCAC	P14-22-2F	AGTTCCAGCTTTTTCTGAAGAC			ACP-Tel		UNI-ACP		
**P15/P16**	LR-PCR	P15-22-F	CCCCTCCAGAGACCCAAAGA					P15-12-R	GCAGCGCCACCTTCCTAAA			
**P20 BP1**	ACP-Tel	P20-22-1F	CCAGGTGCAGTTCCTACAA	P20-22-2F	TGGCTTGAACAGCTCCTCACT			ACP-Tel		UNI-ACP		
**P20 BP2**	ACP-Tel	P20-22-5F	TGTTTACCAAATCCACTCACTGCTT	P20-22-6F	CAGCAATAGATTCCACTCTTAG			ACP-Tel		UNI-ACP		
**P20 BP3**	ACP-Tel	P20-22-3F	TGTTTACCAAATCCACTCACTGCTT	P20-22-4F	CAGCAATAGATTCCACTCTTAG			ACP-Tel		UNI-ACP		
**P21**	ACP-Tel	P21-22-1F	GTGTCTCCCATTTCTCATAAC	P21-22-2F	GTTGGTTCCTCTTGGTCTTG			ACP-Tel		UNI-ACP		
**P26**	Inv-PCR	P26-22-1F	ATGAGGGCTCTGTCAGGCAT	P26-22-2F	TGGATGCAAAGTCCAGTTGA			P26-22-1R	GACTGCGGGCATCCTGGAA	P26-22-2R	CGTGGAGCCGTCTTTGTAAG	
**P30**	ACP-Tel	P30-22-1F	TCAGCTCCCATCCCAAGTG	P30-22-2F	GGGATGGCTAACTGGTCACT			ACP-Tel		UNI-ACP		
**P31**	ACP-Tel	P31-22-1F	GTGCTGTTGTGCTCGGATGGT	P31-22-2F	CTGTGCTCGTGTCCTGCTCTT			ACP-Tel		UNI-ACP		
**P32**	ACP-Tel	P31-22-1F		P31-22-2F				ACP-Tel		UNI-ACP		
**P34**	ACP-Tel	P34-22-1F	GTCAGGAGGCAGAACTTTCA	P34-22-2F	AGGGCATTTCAAGAACCATTTC			ACP-Tel		UNI-ACP		
**P36**	ACP-Tel	P36-22-1F	CTGTTGTGTCTGTTCAGTCCT	P36-22-2F	TGAAGCTGGAGCAGACTATC			ACP-Tel		UNI-ACP		
**P37**	LR-PCR	P37-22-F	ACCCGGTGAATGAGCAAATG					P37-22-R	GCCCATGAAGAGGCTGTTGA			
**P38**	LR-PCR	P38-22-F	AGCGGGAAGGTTAAAAACCAA					P38-22-R	AGGCCGTCTTGGAACAAGTCT			
**P39**	ACP-Tel	P31-22-1F		P31-22-2F				ACP-Tel		UNI-ACP		
**P40**	ACP-Tel	P40-22-1F	GCCGTCAGGCCCAGTCT	P40-22-2F	CAGGGCCTGGGAAGATCAG			ACP-Tel		UNI-ACP		
**P42**	LR-PCR	P42-22-F	TGCCTCCCAAGCCCAAGCTCAAGTC					P42-22-R	ACCTCAGACCCATCCAGTCGATGCC			
**P43**	LR-PCR	P43-22-F	CTCCACCCAGGCCTGTATATTT					P42-22-R				
**P44**	LR-PCR	P44-22-F	CACCACTGTGGCATTCTCTC					P44-22-R	CTGCTTCCTTGCCTCTGTCA			
