# Supplementary material for: Molecular Mechanisms Generating and Stabilizing Terminal 22q13 Deletions in 44 Subjects with Phelan/McDermid Syndrome
Source: PLoS Genet. 2011 Jul 14;7(7):e1002173. doi: 10.1371/journal.pgen.1002173 (PMC3136441; doi:10.1371/journal.pgen.1002173)
Supplement: Text S1 — Supplementary Clinical Information. (PDF) [file pgen.1002173.s009.pdf]

Supplementary Clinical Information

Clinical medical history of adult patients

Subject P30

The patient is a woman referred for clinical genetics evaluation at the age of 40 years because of severe cognitive impairment and mild craniofacial dysmorphisms.
She showed tall stature (>97th centile), in agreement with the stature of other family members; weight and OFC were at the 50th centile. She suffered from epilepsy, cortical tremor (starting at the age of 39 years) and poor speech. Minor facial dysmorphic features were observed(Figure 1A-E). Decreased perception of pain was described.
The first clinical observation, performed at the age of 3 years, reported global psychomotor development delay, lumbar lordosis with cervical scoliosis, left ptosis, mild diffuse hypertonia and absent language (Brunet-Lézine Score**:** E.S.M.=648 ; Q.S.M.= 0.63). Audiometric evaluation was normal. Complex partial seizures occurred at 5 years of age and generalized tonic-clonic seizures at 8 years of age. Two independent karyotype analyses on peripheral blood gave normal results.

Subject P33

The patient is a male first referred to a geneticist at the age of 41 years in the context of familial genetic counseling.
He was 160 cm tall, weighted about 62 kg, and had OFC of 52.5 cm (>97th centile).
The dysmorphological examination revealed evident aspecific dysmorphisms (Figure 1K-N).
He presented with total absence of language, severe mental retardation, delayed motor development and microcephaly. He seemed to understand more than he could express, and had a calm, friendly and compliant behaviour. He had been born at term by spontaneous delivery and was kept in an incubator for a few days because of low birth-weight (2,600 g). His parents reported hyporeflexia but denied neonatal hypotonia or feeding problems. At the age of 4 months, he suffered from viral pneumonia. His mother noticed since the first months of his life that he had an absent look and lacked the ability of tracking and fixing objects. He walked alone at 18 months, with frequent falls. At the age of 2 years, he showed absence of language and delayed motor skills. At the age of 34 years, he developed type 2 diabetes, well compensated by oral hypoglycemic therapy, and had three spontaneous pneumothorax episodes at the upper lobe of his left lung. He has never undergone abdominal ultrasound, echocardiography, cerebral MRI or neuropsychological evaluation. A karyotype was requested at the age of 41 years and a diagnosis of 46, XY, r (22) [110]. ish r (22) (TUPLE1 + N85A3-) was made. His parents' karyotypes were normal.

Subject P10

This female subject was referred to a geneticist at the age of 40 years in the context of a diagnostic evaluation of people living in an institution for mentally disabled people.
Physical examinations showed macrocephaly (>97th centile) while height and weight were in the normal range. Long fingers and relatively long feet, dysplastic toenails, proximal placement of thumbs and bilateral 2nd-3rd toe syndactyly were noticed. Facial dysmorphisms were also evident (Figure 1F-J). Her social relationship was limited to emotional manifestations; communication could occur through eye contact, mimicking and gestures. Her verbal communication was limited to repetitive and guttural vocal sounds apparently not directed to other people. Mental retardation was classified as severe. Neurological evaluation showed spastic paraparesis, with upper limbs maintained in a flexed and abducted position and lower limbs flexed at the knees, with inward

turning feet, while standing. She showed decreased sensitivity to pain and tactile stimuli. She had begun to walk at the age of 3 years. At the same age, she experienced her first epileptic seizure; EEG showed abnormal focal electric activity. Her second seizure occurred at the age of 34 years. At age 39, epileptic seizures became more frequent. EEG recording showed low amplitude, dysrythmic and slow electric activity. Within the following 3 years, in spite of antiepileptic drugs, crises arose in quick succession and lasted for days. At age 43, she experienced very fast motor and cognitive decline; as a consequence, she was not able to stand, walk or even make eye contact anymore; her spastic tetraparesis markedly increased. Right renal agenesis was diagnosed during a control abdominal ultrasonography. She died at 47 years for renal failure while in a vegetative state.
She was born after term through an uncomplicated vaginal delivery and following an uneventful pregnancy. She was the fifth of six siblings; her two sisters and three brothers were healthy and, except for one brother, they all had children, who were healthy as well. Family history was non-contributory. Her mother noticed “something wrong” with her lower limbs when she was about 12 months old; before then, development had been normal. Apart from telling “Ciao” (Hello), she was never able to speak even in single words/word approximations, but her mother could remember she used to approximate her sibs’names. Until she was 5 year-old there were no evident dysmorphisms and her build appeared slender. Scoliosis was documented since the age of 12 years. She has been institutionalised since she was 12 year-old (see Figure 1F). Pubertal development and menarche were unremarkable. Her heart was never evaluated trough ultrasonography, but ECG and cardiological assessment were normal. Her karyotype was 46,XX. During genetic consultation for the 22q13 deletion, her mother declined genetic testing; her father was deceased.

Patients with cryptic interstitial 22q13.3 deletions disrupting the *SHANK3* gene

Subject P37

The patient is a girl, the second child of non-consanguineous parents with unremarkable family history. At birth, her weight was normal; length and OFC were unreported; she presented with cyanosis. Psychomotor developmental milestones were normal, but speech was delayed. At last evaluation, at the age of 23 years, her phenotype was characterized by profound mental retardation, abdominal hypotonia, behavioral disturbances, flat occiput, high palate, pes planus-valgum. Formal tests for autism/ASD were not performed. Other features such as hypothyroidism, hepatomegalia, tremors, and tics were also observed.

Subject P38

This female patient is the second child of non-consanguineous parents with unremarkable family. She was born at term after a pregnancy characterized by poor foetal movements. At birth, her weight was 3550 g (75th-90th), length and OFC were unreported; she also showed cyanosis. Psychomotor development was slightly delayed. At the age of 8 years, her phenotype was characterized by moderate mental retardation, ASD, short stature (H: <3rd; W: 25-50th; OFC: 25th–50th), bilateral astigmatism and some facial dysmorphisms, such us flat occiput, short forehead, downslanting palpebral fissures, anteverted ears. Brain MRI revealed lateral ventricles asymmetry (R>L) and white matter anomalies.

Subject P42

This male subject is the youngest of three siblings born to non-consanguineous parents with unremarkable family history. His older brother is in good health, while the second child, a girl, was stillborn. He was born at term by induced delivery; his weight was 3,750 g, length 55 cm, and OFC

55 cm . From the age of three, the child experienced a profound delay in language acquisition and mild motor delay. At age 6, brain MRI showed a slight dilation of Virchow-Robin perivascular spaces. At age 8, specific testing led to a diagnosis of mild mental retardation (F70 ,ICD10) and excluded autism (CARS, ABC). At age $12^{4/12}$, his weight was 56 Kg (90th centile), height 166.5 cm (97th centile), OFC 56.2 cm (>97th centile, +2SD). He showed elongated face, bilateral spacing between I and II toes, right foot fourth digit clinodactyly. ECG was normal. He could neither read nor write, and only in the last year he had started recognizing some printed letters.
Karyotype and subtelomere FISH screening were normal.

Subject P43

She is the first child born to unrelated parents. Her mother has an autoimmune liver disorder and has undergone liver transplant. She has two half siblings on the paternal side, and one half brother has autism and learning difficulties. In addition, a cousin of her father has developmental problems. During pregnancy a defect in the addominal wall with protusion of guts was detected by ultrasound. She was therefore delivered by caesarian section, and early surgical intervention was successful. She has developed a classic autism in combination with hyperactivity disorder. There are no dysmorphic features, and growth is within the normal range (length and weight –1 SD)
Genetic evaluation was started at age 5, when karyotype and fragile X revealed normal results. Array-CGH disclosed a small deletion on 22q13.3 in addition to a 400 kb duplication on 9p24.3. None of the aberrations were present in her father or her affected half brother. The duplication on 9p is most probably a normal variant since her mother is a carrier.
The deletion on 22q13.3 was further investigated with FISH (four terminally located cosmid probes) confirming the location of the deletion.

Subject P44

This male subject was adopted at the age of 2 years and 10 months. He was born in Colombia; family history, perinatal history and psychomotor developmental milestones are unknown until the age of adoption. At the age of 2 years and 10 months the child had a profound delay in language acquisition (no word was pronounced even in the original language). When he first came to our attention at 6 years of age, at general examination his weight was 19 Kg (50th centile), height was 115 cm (50th centile); microcephaly was present (OFC: 49.3 cm, <3rd centile). He presented skin fragility with delayed wound healing. On formal psychological assessment using the Griffiths scale he was noted to have moderate mental retardation with particular involvement of expressive language and good interpersonal skills. Brain MRI and electrophysiological tests (Electroencefalography, Brainstem Auditory Evoked Potentials) were normal. Ophthalmological evaluation revealed astigmatism and normal fundus oculi.

Subjects with atypical clinical features

Subject P19

At birth, the baby showed ambiguous genitalia with shawl scrotum, micropenis and hypospadias associated to mild hypotonia, minor dysmorphic features (flat phyltrum and flat occiput) and cardiac anomalies (ASD and VSD). Karyotype analysis was normal, while subtelomeric-FISH analysis revealed pure 22q13.3 monosomy. Whole-genome aCGH analysis, performed to size the 22q deletion, excluded the presence of further pathogenic imbalances interspersed through the genome. Since ambiguous genitalia is a feature never reported in association with 22q distal

deletion, it is most likely caused by independent mutation of a gene involved in the sex differentiation pathway.
